# A Symmetry-Reduced Differentiable Neural Information Field for Geometric Angle-Only Trackability Assessment Across Walker Low-Earth-Orbit Sensor Constellations

**DOI:** 10.3390/s26185902

**Published:** 2026-09-17

**Authors:** Hengguo Zhang, Kebo Li, Yangang Liang, Yunxiao Lv

**Affiliations:** 1 College of Aerospace Science and Engineering, National University of Defense Technology, Changsha 410073, China; zhanghengguo18@nudt.edu.cn (H.Z.); liangyg@nudt.edu.cn (Y.L.); 2 College of Intelligence Science and Technology, National University of Defense Technology, Changsha 410073, China

**Keywords:** angle-only sensing, Fisher information, Walker constellation, neural information field, symmetry reduction, *J*_2_ perturbation, sensor reconfiguration

## Abstract

Persistent angle-only tracking with Walker low-Earth-orbit constellations requires repeated geometric assessment, but optimal sensor-subset selection is computationally costly. To support efficient assessment, a symmetry-reduced differentiable neural information field is proposed to approximate the A-optimal potential of the best feasible sensor subset of a prescribed size. Its two-stage network replaces absolute time with bounded phases derived from axial and finite-permutation symmetries of a relative-periodic Walker constellation under $J_{2}$. For a 25×25 constellation, symmetry reduction decreased the three-seed mean root-mean-square error from $7.664\times 10^{-3}$ to $1.154\times 10^{-3}$ relative to a matched two-body encoding; altitude-gradient correlation reached 0.9960. Finite-symmetry closure was verified for Delta, Star, and Rosette. Field accuracy remained within prescribed thresholds for 30 days under matched $J_{2}$ dynamics. Without orbit maintenance, high-fidelity propagation yielded median and minimum sampled validity horizons of 1.50 and 1.25 days. On historical Iridium trajectories reconstructed from orbital records, three independently trained fields maintained the prescribed accuracy for at least 30 days, demonstrating long-horizon applicability to an operational Walker constellation. Neural triggering reduced penalized mean regret by 91.6% and candidate-pool searches by 93.4%. Full-grid neural inference achieved 17.5-fold CPU and 5567-fold GPU speedups over CPU candidate-pool evaluation.

## 1. Introduction

Space-based optical sensors provide line-of-sight angular measurements for target surveillance, angles-only orbit determination, and cooperative spacecraft observation and navigation [1,2,3,4]. These applications require informative observation geometry as the relative positions of sensors and targets evolve. For a low-Earth-orbit (LEO) sensor constellation observing high-altitude moving targets, geometric assessment supports the identification of favorable observation regions and the reassignment of sensors along a target trajectory. These tasks require efficient assessment of how observation geometry varies across target locations and over time.

Across these tasks, geometric visibility is necessary but not sufficient for informative angle-only measurements. Simultaneously visible satellites can provide nearly parallel lines of sight, leaving position information strongly anisotropic. Fisher information criteria quantify this effect in angle-only path planning, sensor selection, and cooperative orbital sensing [3,5,6]. Applying these criteria over large constellations and extended trajectories requires repeated assessment at many target locations and times. Direct evaluation at each query requires satellite positions, visibility screening, and the construction of Fisher information matrices (FIMs), followed by a search over admissible sensor subsets [7,8,9]. The resulting computational cost limits dense mapping and repeated tasking, while the individually evaluated samples do not directly provide a continuous field or its spatial gradients.

Existing approaches address individual parts of this computational chain. Reinforcement learning, tree search, potential games, and mathematical scheduling support sensor selection and tasking, but still require repeated evaluation of geometry or uncertainty [10,11,12,13,14]. Constellation design studies similarly couple coverage with observation geometry [1], while optimization methods address constellation design decisions [15]. Fisher information fields organize sensing geometry as a reusable spatial representation for static visual localization [16], while neural surrogates reduce repeated computations in constellation analysis [17]. Coordinate-based neural fields with Fourier features provide continuous pointwise queries and spatial derivatives [18,19,20,21], and physics-informed models incorporate known structure into learned mappings [22,23]. Neural orbit models have focused on two-body solutions and orbital-element prediction [24,25]. For a neural information field of an orbiting sensor constellation, a further issue is how to represent temporal evolution while exploiting the relative periodicity of the sensing geometry.

For a Walker constellation [26], the informative geometry changes jointly with target longitude, latitude, altitude, Earth rotation, orbital phase, and inter-plane phasing. Using absolute time as an additional network coordinate combines these motions in an unbounded variable and turns long-term evaluation into temporal extrapolation. The $J_{2}$ perturbation adds nodal precession and short-period variation to the ideal Keplerian geometry [27,28]. These dynamics motivate a symmetry-based temporal representation, together with an assessment of how long the resulting field remains accurate when additional perturbations are present.

This study develops a symmetry-reduced neural information field for assessing the observation geometry of Walker LEO sensor constellations, with numerical validation focused on moving targets at altitudes of 15–100 km. The reference quantity is the A-optimal potential $\psi_{A}$, minimized over feasible sensor subsets of a prescribed size within a range-ranked candidate pool. Smaller values indicate more informative local geometry. The reported implementation and experiments use three-satellite subsets. With standard $J_{2}$ dynamics defining the nominal constellation model, the proposed reduction uses axial equivariance and a topology-dependent finite permutation to map absolute time to bounded co-precessing and fast phases. A two-stage coordinate network then approximates $\psi_{A}$ over target longitude, latitude, altitude, and phase, providing differentiable potential estimates for feasible queries within the sampled domain. Separate tests quantify neural approximation error and field validity under matched and high-fidelity dynamics and on historical operational trajectories.

The principal contributions are threefold:

1. A symmetry-reduced representation maps Earth rotation, $J_{2}$ nodal precession, and topology-specific Walker slot recurrence to bounded co-precessing and fast phase coordinates for the angle-only information field.
2. A two-stage differentiable surrogate provides efficient pointwise estimates of the candidate-pool potential $\psi_{A}$ and its spatial gradients for geometric assessment.
3. The validity analysis distinguishes neural approximation error from dynamics–model mismatch and quantifies field validity under uncontrolled high-fidelity dynamics and along historical Iridium operational trajectories. An application demonstration evaluates field-guided search triggering for sensor reconfiguration.

## 2. Theory and Symmetry Reduction

The angle-only information field is determined jointly by sensing geometry and constellation dynamics. This section first formulates the angle-only information measure and a nominal Walker constellation under standard $J_{2}$ dynamics. The time-dependent field is then represented on a bounded phase domain using the constellation’s axial and finite-permutation symmetries, without requiring the field itself to be smooth.

### 2.1. Problem Definition and Reference Frames

A field query is specified by the Earth-fixed geocentric longitude $\lambda_{E}$, geocentric latitude φ, altitude *h*, and time *t*. The vector $r_{T}^{E}$ denotes the geocentric position of the query target, measured from the Earth center *O* and resolved along the axes of the adopted Earth-fixed frame $F^{E}$. Under the spherical-Earth approximation, the target position is given by

(1) $$r_{T}^{E}\left(\lambda_{E},\varphi ,h\right)=\left(R_{E}+h\right)\left[\begin{array}{c} \cos\varphi \cos\lambda_{E}\\ \cos\varphi \sin\lambda_{E}\\ \sin\varphi \end{array}\right],$$

where $R_{E}$ is the reference Earth radius and *h* is the radial altitude above the reference sphere. Longitude $\lambda_{E}$ is measured in the equatorial plane from the positive $x^{E}$ axis toward the positive $y^{E}$ axis.

Let $t_{0}$ be the reference epoch, $\Delta t=t-t_{0}$, and $\theta_{G,0}$ the Earth rotation angle at $t_{0}$. The adopted constant-rate Earth rotation model is

(2) $$\theta_{G}\left(t\right)=\theta_{G,0}+\omega_{E}\Delta t,\;r^{E}\left(t\right)=R_{z}\left[-\theta_{G}\left(t\right)\right]r^{I}\left(t\right).$$

**Figure 1:**
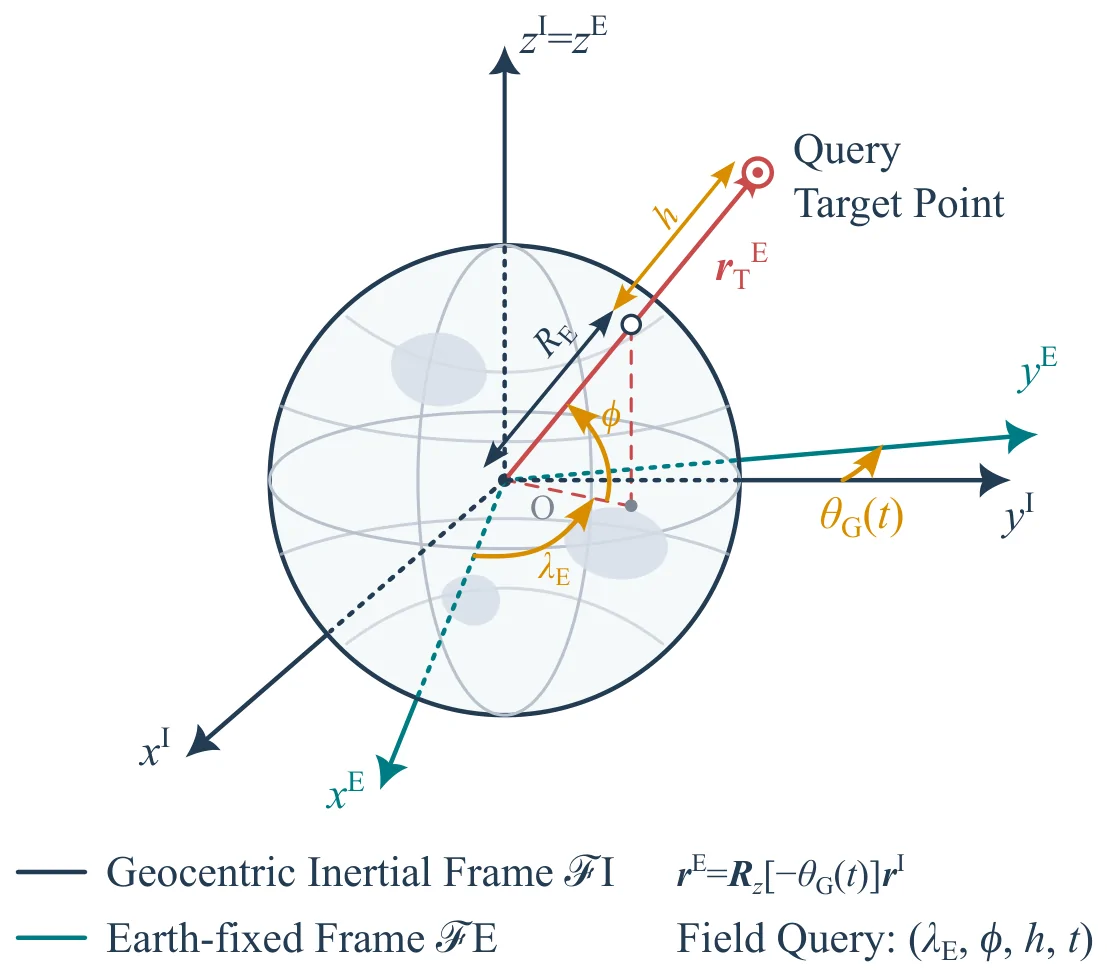
Reference frames and target coordinates for an information-field query.

Here, $\omega_{E}$ is the Earth rotation rate, and the superscript I denotes the geocentric inertial frame $F^{I}$. The angle $\theta_{G}\left(t\right)$ describes the positive rotation of the Earth-fixed axes relative to the inertial axes about their common *z*-axis. With the active-rotation convention for $R_{z}$, the inertial-to-Earth-fixed component transformation uses $-\theta_{G}\left(t\right)$. Figure 1 illustrates the reference frames and target coordinates.

**Figure 2:**
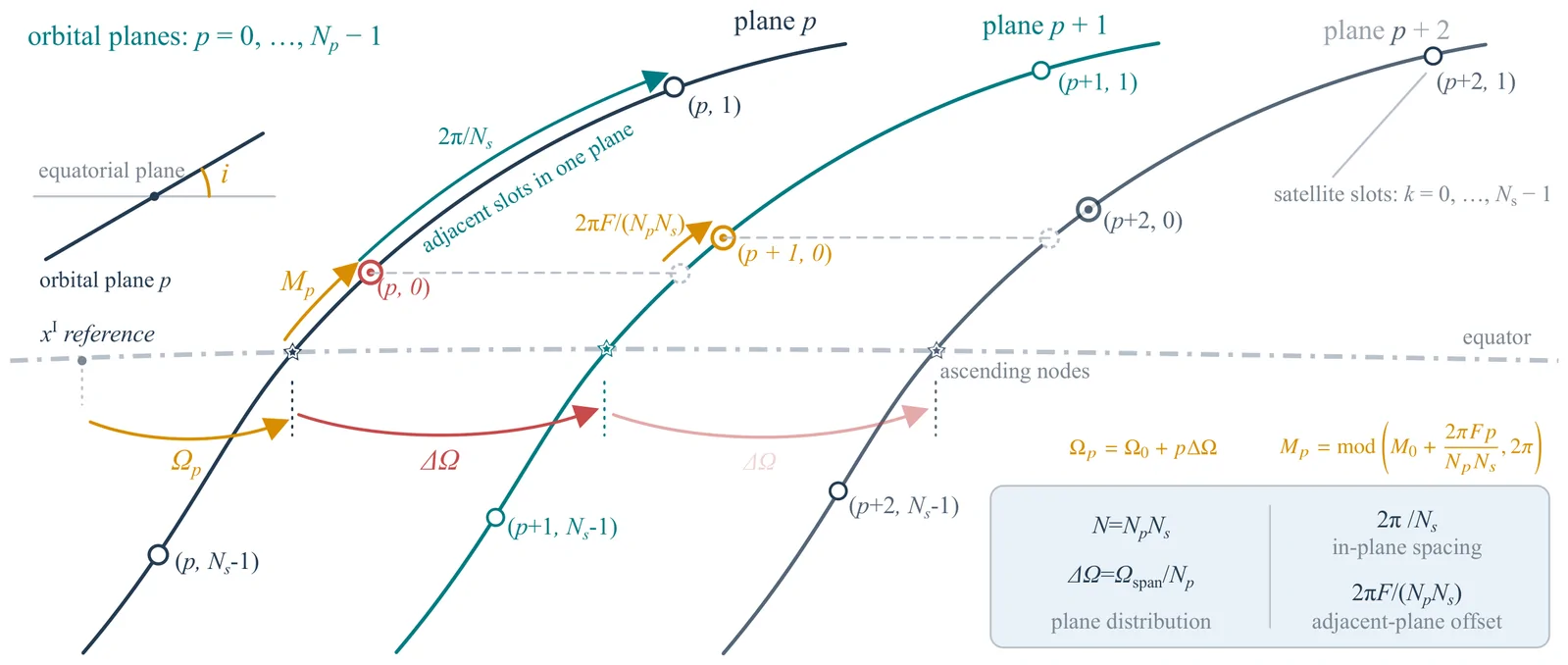
Walker orbital-plane and slot parameters at the reference epoch.

Consider a Walker-type LEO sensor constellation with $N_{p}$ orbital planes and $N_{s}$ satellites per plane, giving $N=N_{p}N_{s}$ sensors in total [26]. The plane and in-plane slot indices are $p\in \{0,\ldots ,N_{p}-1\}$ and $k\in \{0,\ldots ,N_{s}-1\}$, respectively. At the reference epoch, the node and phase relations shown in Figure 2 are

(3) $$\begin{array}{cccc} \Omega_{p} & =\Omega_{0}+p\Delta \Omega ,\; & \;\Delta \Omega & =\frac{\Omega_{\mathrm{span}}}{N_{p}},\\ M_{p} & =\mathrm{mod}\;\left(M_{0}+\frac{2\pi Fp}{N_{p}N_{s}},2\pi \right),\; & M_{p,k} & =\mathrm{mod}\;\left(M_{p}+\frac{2\pi k}{N_{s}},2\pi \right). \end{array}$$

Here, $\Omega_{0}$ is the reference right ascension of the ascending node (RAAN), $M_{0}$ is the reference along-track phase, $\Omega_{\mathrm{span}}$ is the node span, ΔΩ is the adjacent-plane node spacing, and *F* is the Walker phasing parameter. The orbital size is common to all slots, and *i* is the common inclination. Thus, $2\pi /N_{s}$ is the separation between adjacent slots in one plane, whereas $2\pi F/(N_{p}N_{s})$ is the phase offset between the reference slots of adjacent planes. These relations describe the conventional Walker geometry; Section 2.3 constructs the corresponding common-epoch states on a numerically closed $J_{2}$ reference orbit.

At time *t*, the propagated satellite positions are denoted by $r_{i}^{E}\left(t\right)$, i=1,…,N. Let P collect the fixed orbital, frame, Earth-rotation, sensing, and normalization parameters. The reference model maps a query to an information potential and a feasibility indicator,

(4) $$\Psi_{\mathrm{ref}}:\left(\lambda_{E},\varphi ,h,t;P\right)⟼\left(\psi_{A},f\right).$$

The parameter set P includes the Walker geometry, the relative-periodic reference orbit, the frame convention, and the sensing constraints. The potential $\psi_{A}$ and feasibility indicator *f* are defined below.

### 2.2. Angle-Only Information Potential

The reference calculation first determines which satellites can provide admissible angle measurements. For satellite *i*, define the target-to-sensor relative position, range, and unit line-of-sight vector by

(5) $$\Delta r_{i}^{E}\equiv r_{i}^{E}-r_{T}^{E},\;\rho_{i}\equiv {∥\Delta r_{i}^{E}∥}_{2},\;\ell_{i}\equiv \frac{\Delta r_{i}^{E}}{\rho_{i}}.$$

Earth occlusion is evaluated on the complete target-to-sensor segment. Its minimum geocentric distance is

(6) $$d_{i}^{\mathrm{seg}}=\min_{0\leq u\leq 1}{∥r_{T}^{E}+u\Delta r_{i}^{E}∥}_{2}.$$

For the reference field used in this study, the visible set is

(7) $$V=\left\{i:\rho_{i}\leq \rho_{\max},\;d_{i}^{\mathrm{seg}}>R_{E}+c_{E}\right\},$$

where $\rho_{\max}$ is the maximum sensing range and $c_{E}$ is the required Earth clearance.

The measurement model assumes locally isotropic, independent angular errors with standard deviation $\sigma_{\theta }$ in the two-dimensional tangent plane of each line of sight. Linearization with respect to the target position gives the single-sensor Fisher information matrix (FIM)

(8) $$J_{i}=\frac{1}{\sigma_{\theta }^{2}\rho_{i}^{2}}\left(I_{3}-\ell_{i}\ell_{i}^{T}\right).$$

The projection matrix in Equation (8) contributes information perpendicular to the line of sight but none along it. The range-dependent weight $w_{i}={\left(\sigma_{\theta }^{2}\rho_{i}^{2}\right)}^{-1}$ therefore favors nearby measurements, and the sum of several FIMs captures whether their viewing directions complement one another. This use of Fisher information is consistent with information-based angle-only sensing and sensor-selection formulations [5,6].

The reference criterion is defined on a range-weighted candidate set. Let $N_{\mathrm{sel}}\geq 2$ denote the prescribed number of selected sensors; the implementation and all experiments reported here use $N_{\mathrm{sel}}=3$. The visible sensors are ranked by $w_{i}$, and

(9) $$K={\mathrm{Top}}_{K_{0}}\left(V;w_{i}\right)$$

contains the $K_{0}$ largest-weight sensors, or all visible sensors when $|V|<K_{0}$. For a subset C⊆K with $\left|C\right|=N_{\mathrm{sel}}$, the combined information matrix is

(10) $$J_{C}=\sum_{i\in C}J_{i}.$$

Only subsets satisfying $\mathrm{rcond}\left(J_{C}\right)\geq r_{\min}$ are retained, where $r_{\min}$ is a reciprocal-condition-number threshold. The selected set is

(11) $$C^{★}=\underset{\begin{array}{c} C\subseteq K,\;|C|=N_{\mathrm{sel}}\\ \mathrm{rcond}\left(J_{C}\right)\geq r_{\min} \end{array}}{\arg\min}\;\mathrm{tr}\left(J_{C}^{-1}\right).$$

Equation (11) considers every $N_{\mathrm{sel}}$-sensor subset in the candidate set K. The resulting optimum is therefore exact within K, although it need not equal the global optimum over all visible satellites. The criterion uses the unregularized matrix $J_{C}$; subsets that fail the reciprocal-condition threshold or yield a nonfinite or nonpositive value of $\mathrm{tr}(J_{C}^{-1})$ are excluded from the feasible set.

The square root of the A-optimal criterion has units of distance and summarizes the three principal position-uncertainty directions. The dimensionless information potential is defined as

(12) $$\psi_{A}=\log_{10}\left[\frac{\sqrt{\mathrm{tr}\left(J_{C^{★}}^{-1}\right)}}{d_{0}}\right],\;d_{0}=1\;\mathrm{km}.$$

The fixed reference length $d_{0}=1\;\mathrm{km}$ renders the logarithm dimensionless and provides a common scale for interpreting the A-optimal position-uncertainty measure. Replacing $d_{0}$ with any other fixed length would only add a constant offset to $\psi_{A}$, leaving sensor-subset rankings, spatial gradients, and potential differences unchanged. Keeping $d_{0}$ independent of sensing parameters ensures consistent normalization across sensing configurations.

A smaller $\psi_{A}$ denotes a more informative local geometry with a smaller A-optimal uncertainty scale. If fewer than $N_{\mathrm{sel}}$ sensors are visible or no candidate subset satisfies the feasibility conditions, f=0 and $\psi_{A}$ is undefined; otherwise, f=1. The quantity $\psi_{A}$ is a local geometric information proxy and does not directly predict filtering root-mean-square error (RMSE), which also depends on target dynamics, prior uncertainty, measurement timing, and estimator design.

**Figure 3:**
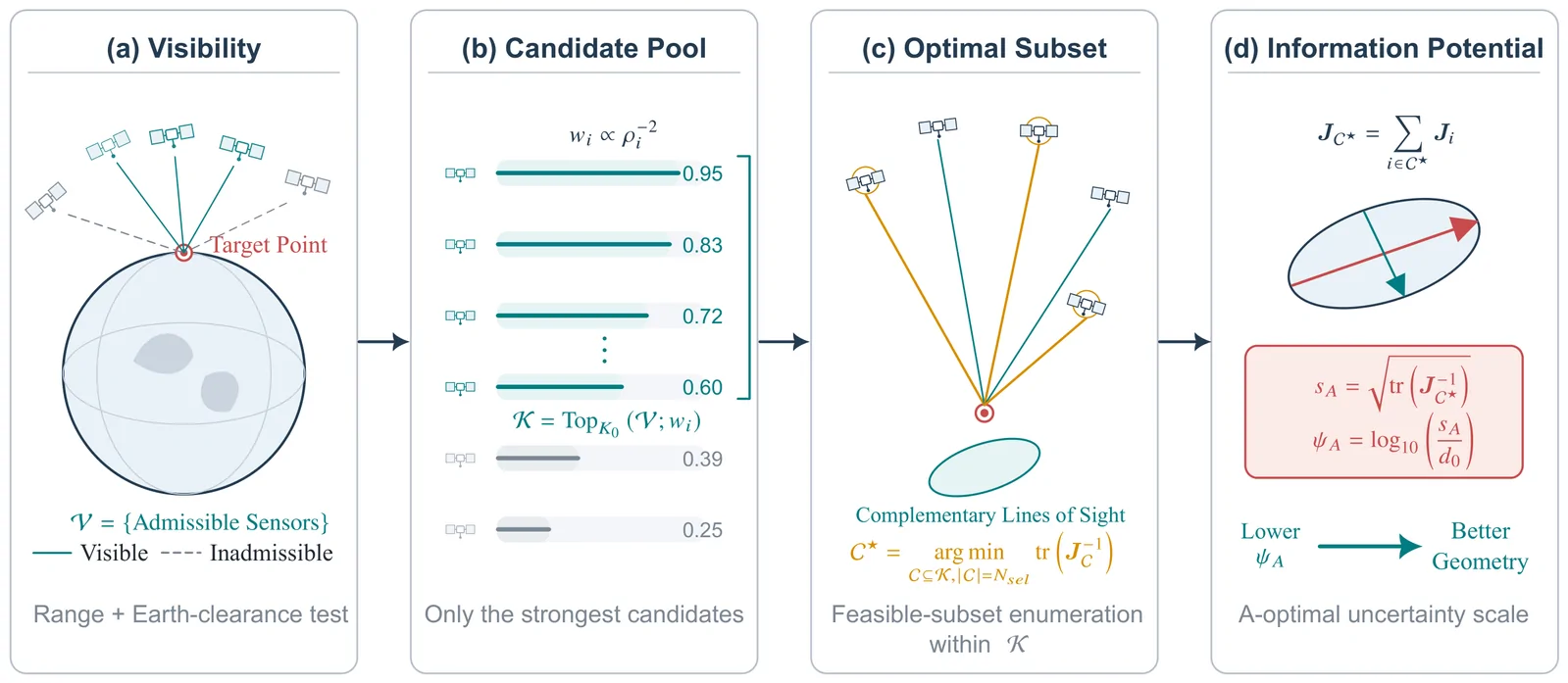
Angle-only information-potential construction.

Figure 3 summarizes the construction from visibility screening to the A-optimal information potential.

### 2.3. Relative-Periodic Walker Constellation Under $J_{2}$ Perturbation

The nominal constellation model combines the established Walker parameterization [26] with a conventional orbital-dynamics model comprising central-body gravity and the $J_{2}$ perturbation [28]. The subsequent information-field reduction exploits the axial equivariance of the dynamics and the finite-permutation symmetry of the constellation.

A Walker constellation is defined by repeated orbital planes and prescribed along-track phase offsets applied to a common orbit. Under an oblate gravity field, independently assigning two-body circular states at different argument latitudes does not preserve this common-orbit relation because the $J_{2}$ potential varies with latitude. The corresponding Walker model is instead formed from phase-shifted states of one orbit that is periodic in a uniformly co-precessing frame. The adopted inertial dynamics are

(13) $$\ddot{r}=-\frac{\mu }{r^{3}}r+\frac{3\mu J_{2}R_{E}^{2}}{2r^{5}}\left[\begin{array}{c} x\left(5z^{2}/r^{2}-1\right)\\ y\left(5z^{2}/r^{2}-1\right)\\ z\left(5z^{2}/r^{2}-3\right) \end{array}\right].$$

Here, $r={\left[x,y,z\right]}^{T}$, $r={∥r∥}_{2}$, and μ is the gravitational parameter. The first term in Equation (13) is the central-body acceleration, and the second is the $J_{2}$ perturbation. This central-gravity-plus-$J_{2}$ system is the nominal axisymmetric model used to construct the relative-periodic Walker geometry and bounded phase representation; it is not a complete high-fidelity model of LEO motion [27]. Higher Earth-gravity harmonics, third-body gravity, solar radiation pressure, and atmospheric drag are treated as sources of dynamics–model mismatch in Section 4.5. Geometry-altering maneuvers likewise fall outside the analytical symmetry relation.

Let $\Phi_{\Delta t}$ denote the state flow of Equation (13), and define the simultaneous rotation of position and velocity by

(14) $$G\left(\gamma \right)\left[\begin{array}{c} r\\ v \end{array}\right]=\left[\begin{array}{c} R_{z}\left(\gamma \right)r\\ R_{z}\left(\gamma \right)v \end{array}\right].$$

Because the dynamics are autonomous and axisymmetric,

(15) $$\Phi_{\Delta t}\;\left(G(\gamma )x\right)=G\left(\gamma \right)\Phi_{\Delta t}\left(x\right).$$

A reference state $x_{★}$, a return time $T_{\mathrm{rp}}$, and a nodal rotation $\Delta \Omega_{\mathrm{rp}}$ are sought such that

(16) $$\Phi_{T_{\mathrm{rp}}}\left(x_{★}\right)=G\left(\Delta \Omega_{\mathrm{rp}}\right)x_{★},\;{\dot{\Omega }}_{\mathrm{rp}}=\frac{\Delta \Omega_{\mathrm{rp}}}{T_{\mathrm{rp}}}.$$

Equation (16) describes an orbit that is relative-periodic and not closed in inertial space: after one argument-latitude cycle, the complete state is recovered modulo the common nodal rotation $\Delta \Omega_{\mathrm{rp}}$. When $x_{★}$ is placed on an ascending-node section, $T_{\mathrm{rp}}$ is the return time to the corresponding co-precessing section and $\Delta \Omega_{\mathrm{rp}}$ is the accumulated nodal displacement. These quantities are obtained from the numerical solution of the $J_{2}$ model; the integration and convergence specifications are reported with the experimental protocol.

The periodic trajectory in the co-precessing frame is

(17) $$y\left(q\right)=G\;\left(-q\Delta \Omega_{\mathrm{rp}}\right)\Phi_{qT_{\mathrm{rp}}}\left(x_{★}\right),\;q\in R,$$

for which Equation (16) implies y(q+1)=y(q). This closed curve provides the common orbit from which all Walker slots are defined. For plane $p\in \{0,\ldots ,N_{p}-1\}$ and slot $k\in \{0,\ldots ,N_{s}-1\}$, let

(18) $$q_{p,k}=\mathrm{mod}\left(\frac{M_{0}}{2\pi }+\frac{k}{N_{s}}+\frac{Fp}{N_{p}N_{s}},1\right),\;\Omega_{p}=\Omega_{0}+\frac{\Omega_{\mathrm{span}}p}{N_{p}}.$$

The common-epoch state is then

(19) $$x_{p,k}\left(t_{0}\right)=G\left(\Omega_{p}\right)y\left(q_{p,k}\right).$$

The factor $G(-q\Delta \Omega_{\mathrm{rp}})$ in Equation (17) removes the uniform nodal rotation from the phase-shifted state, while short-period variations remain part of the $J_{2}$ flow Φ. Equation (19) is therefore the $J_{2}$ analogue of placing phase-shifted satellites on a common Walker orbit. The node span $\Omega_{\mathrm{span}}$ equals 2π for a Walker Delta layout and π for a Walker Star layout.

**Figure 4:**
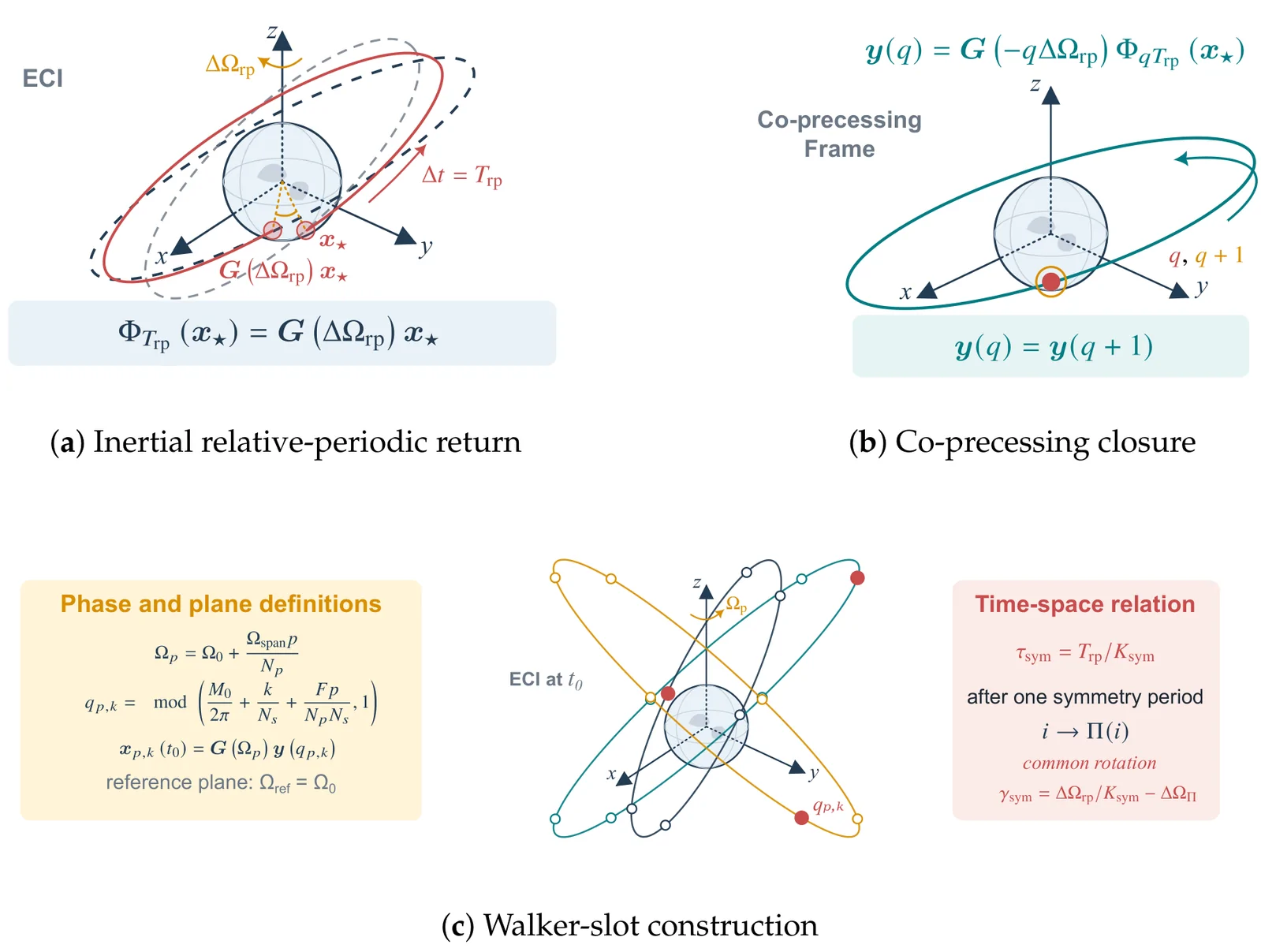
Relative-periodic Walker construction under $J_{2}$ perturbation. In (**a**), the red curve shows propagation over $T_{\mathrm{rp}}$, and the dashed curves indicate the initial and rotated orbital orientations. In (**c**), colors distinguish orbital planes, and red markers highlight representative slots.

Figure 4 illustrates the relative-periodic orbit and the placement of Walker slots on that common orbit.

The panels separate inertial relative-periodicity, co-precessing closure, and topology-dependent slot placement. Figure 4c also introduces the topology-specific finite permutation used in the temporal reduction.

The temporal reduction requires a finite permutation Π of the unlabeled satellite set. Let $K_{\mathrm{sym}}$ be the order of this permutation and let $\Delta \Omega_{\Pi }$ be the common RAAN increment associated with one application of Π. The corresponding symmetry period and common inertial rotation are

(20) $$\tau_{\mathrm{sym}}=\frac{T_{\mathrm{rp}}}{K_{\mathrm{sym}}},\;\gamma_{\mathrm{sym}}=\frac{\Delta \Omega_{\mathrm{rp}}}{K_{\mathrm{sym}}}-\Delta \Omega_{\Pi }.$$

**Figure 5:**
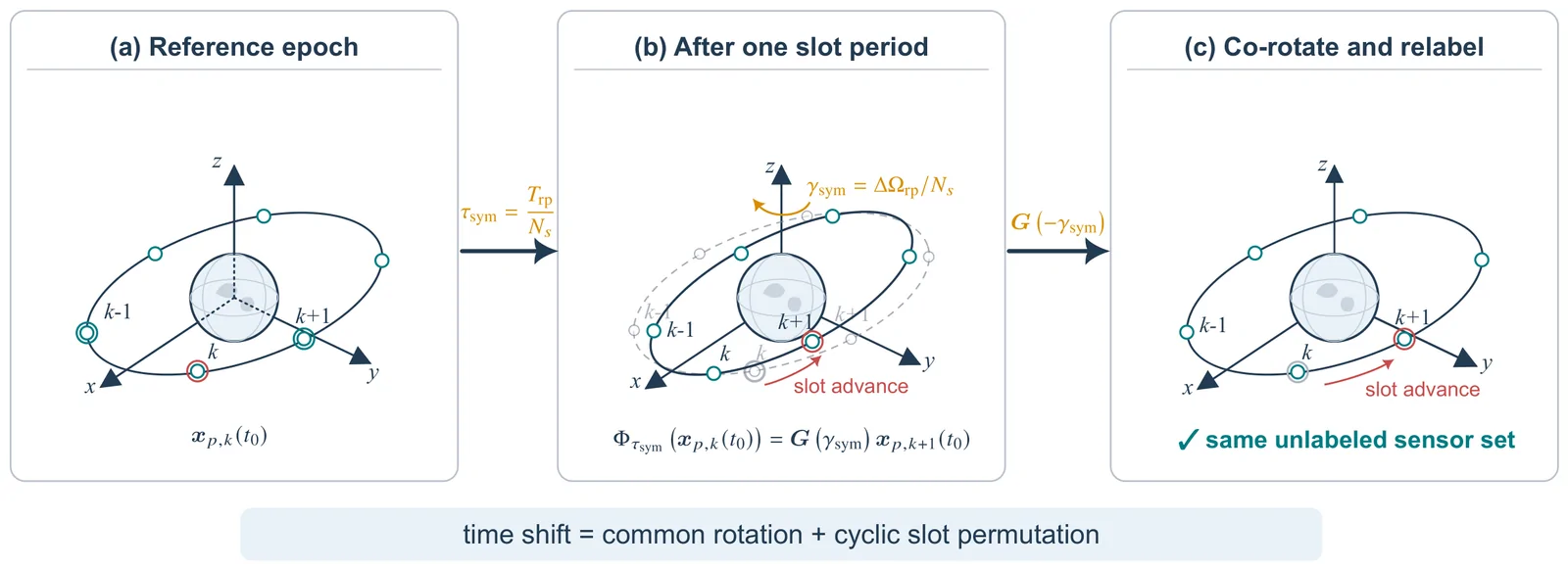
In-plane slot equivariance over one symmetry period.

For any satellite index *i*, Equations (15)–(19) then give

(21) $$\Phi_{\tau_{\mathrm{sym}}}\left(x_{i}\left(t_{0}\right)\right)=G\left(\gamma_{\mathrm{sym}}\right)x_{\Pi (i)}\left(t_{0}\right),$$

where Equation (21) holds only for topologies that admit such a constant-step permutation. For Walker Delta and Walker Star constellations with an in-plane slot cycle, $K_{\mathrm{sym}}=N_{s}$, Π(p,k)=(p,k+1), and $\Delta \Omega_{\Pi }=0$. For the $N_{s}=1$, F=1 inclined rosette constellation considered in Section 4.4.3 [29], $K_{\mathrm{sym}}=N_{p}$, Π(p,0)=(p+1,0), and $\Delta \Omega_{\Pi }=\Omega_{\mathrm{span}}/N_{p}$. Thus, after one symmetry period, the unlabeled sensor set differs from its initial geometry only by the topology-specific permutation and the common inertial rotation $\gamma_{\mathrm{sym}}$. The usual slot equivariance is the in-plane special case of this finite symmetry. Figure 5 depicts the in-plane slot-cycle case, for which $K_{\mathrm{sym}}=N_{s}$, $\Delta \Omega_{\Pi }=0$, and Π(p,k)=(p,k+1).

### 2.4. $J_{2}$ Co-Precessing Symmetry Reduction

The collective rotation of the unlabeled constellation geometry and Earth rotation can be absorbed into a target longitude measured in the constellation’s symmetry-reduced frame. Define the rate of this collective rotation as

(22) $${\dot{\gamma }}_{\mathrm{sym}}=\frac{\gamma_{\mathrm{sym}}}{\tau_{\mathrm{sym}}}={\dot{\Omega }}_{\mathrm{rp}}-\frac{K_{\mathrm{sym}}\Delta \Omega_{\Pi }}{T_{\mathrm{rp}}}.$$

Combining Equation (2) with this finite symmetry gives the two bounded phase coordinates

(23) $$\begin{array}{c} \lambda_{J_{2}}\equiv \lambda_{E}+\theta_{G,0}+\left(\omega_{E}-{\dot{\gamma }}_{\mathrm{sym}}\right)\Delta t-\Omega_{0}\;\left(\mathrm{mod}\;2\pi \right),\\ \alpha_{J_{2}}\equiv K_{\mathrm{sym}}\left(\frac{2\pi \Delta t}{T_{\mathrm{rp}}}+M_{0}\right)\;\left(\mathrm{mod}\;2\pi \right). \end{array}$$

In the first line, $\omega_{E}\Delta t$ transforms the Earth-fixed target into the inertial frame, while $-{\dot{\gamma }}_{\mathrm{sym}}\Delta t$ removes the collective rotation of the unlabeled Walker geometry. For an in-plane slot cycle, $\Delta \Omega_{\Pi }=0$, and $\lambda_{J_{2}}$ reduces to the nodal co-precessing longitude. For a cross-plane Rosette cycle, the plane-spacing term in ${\dot{\gamma }}_{\mathrm{sym}}$ is required because the permutation changes the reference RAAN. The second line represents the remaining finite-permutation evolution through the fast symmetry phase $\alpha_{J_{2}}$. Increasing time by $\tau_{\mathrm{sym}}$ changes $\alpha_{J_{2}}$ by 2π, corresponding to the cyclic relabeling in Equation (21). For fixed physical parameters P, the reference potential can therefore be represented as

(24) $$\psi_{A}\left(\lambda_{E},\varphi ,h,t;P\right)={\tilde{\Psi }}_{\mathrm{ref}}\left(\lambda_{J_{2}},\alpha_{J_{2}},\varphi ,h;P\right),$$

where the first two arguments are elements of the circle and are consequently bounded. The reduction preserves time dependence through two physically interpretable phases: $\lambda_{J_{2}}$ tracks Earth rotation relative to the unlabeled constellation geometry, and $\alpha_{J_{2}}$ tracks the finite satellite permutation.

Because both phases are elements of the circle, sine and cosine coordinates represent them without introducing an arbitrary branch cut. Higher harmonics preserve this periodic representation. Under the stated symmetry assumptions, this mapping converts absolute-time queries into interpolation on a bounded phase domain and avoids extrapolation along an unbounded time coordinate.

### 2.5. Assumptions and Symmetry Boundaries

The exactness of Equation (24) is conditional on the model used to construct it. The principal conditions are as follows:

1. The nominal constellation topology, satellite set, slot assignment, orbital altitude, inclination, and phasing are fixed in the analytical reduction. The effects of small, continuous orbital-element deviations that preserve these discrete attributes are quantified empirically in Section 4.4.4.
2. Satellite motion follows the central-gravity-plus-$J_{2}$ model in Equation (13), and Earth rotation is uniform. Higher gravity harmonics, third-body gravity, solar radiation pressure, atmospheric drag, maneuvers, and timing errors lie outside the analytical guarantee.
3. The target uses spherical geocentric longitude, latitude, and altitude. The learned field is restricted to the sampled altitude interval of 15–100km; intermediate altitudes are interpolation cases, not out-of-range generalization.
4. The reference visibility model includes the range and Earth-clearance conditions in Equation (7); boresight-dependent field-of-view and limb-detection constraints are not modeled. Its hard visibility decisions and discrete subset selection can introduce nonsmoothness when a sensor enters the visible set or two subsets exchange rank.
5. The adopted epoch and frame convention must be transformed explicitly before comparison with an external high-fidelity propagator or ephemerides. A numerical time value alone is insufficient to establish frame and time-scale consistency.

Under these conditions, the analytical reduction bounds the temporal coordinates. Direct evaluation remains expensive because each query entails orbit propagation, visibility testing, FIM construction, and fixed-cardinality subset enumeration. The neural information field therefore approximates the reduced mapping ${\tilde{\Psi }}_{\mathrm{ref}}$.

## 3. Neural Information Field

For fixed P, Equation (24) defines an information field over two periodic coordinates, target latitude, and altitude. The neural information field parameterizes this reduced mapping and enables continuous pointwise evaluation with respect to target location, altitude, and time. The analytical encoder determines the bounded coordinates, and the network approximates the nonlinear relation between the reduced geometry and the information potential.

More precisely, let

(25) $$D=S^{1}\times S^{1}\times \left[-\pi /2,\pi /2\right]\times \left[h_{\min},h_{\max}\right],\;D_{f}=\left\{\xi \in D:f(\xi )=1\right\},$$

where $\xi =(\lambda_{J_{2}},\alpha_{J_{2}},\varphi ,h)$. The potential is defined on $D_{f}$, whereas feasibility is defined on all of D. The network returns a potential estimate at every query, but the estimate is used only when the feasibility output indicates that a feasible sensor subset exists.

### 3.1. Symmetry-Reduced Field Formulation

For a raw Earth-fixed geocentric query $q=(\lambda_{E},\varphi ,h,t)$, the complete model is

(26) $$q\overset{\;E_{J_{2}}\left(\cdot ;P\right)\;}{\to }\left(x_{\mathrm{ctx}},x_{\mathrm{tar}}\right)\overset{\;f_{\Theta }\;}{\to }\left({\hat{\psi }}_{A},{\hat{p}}_{f}\right).$$

The analytical encoder $E_{J_{2}}$ evaluates the two reduced phase coordinates in Equation (23). The neural parameters are denoted by Θ, ${\hat{\psi }}_{A}$ is the potential estimate, and ${\hat{p}}_{f}$ indicates whether the reference query has a feasible $N_{\mathrm{sel}}$-sensor solution.

**Figure 6:**
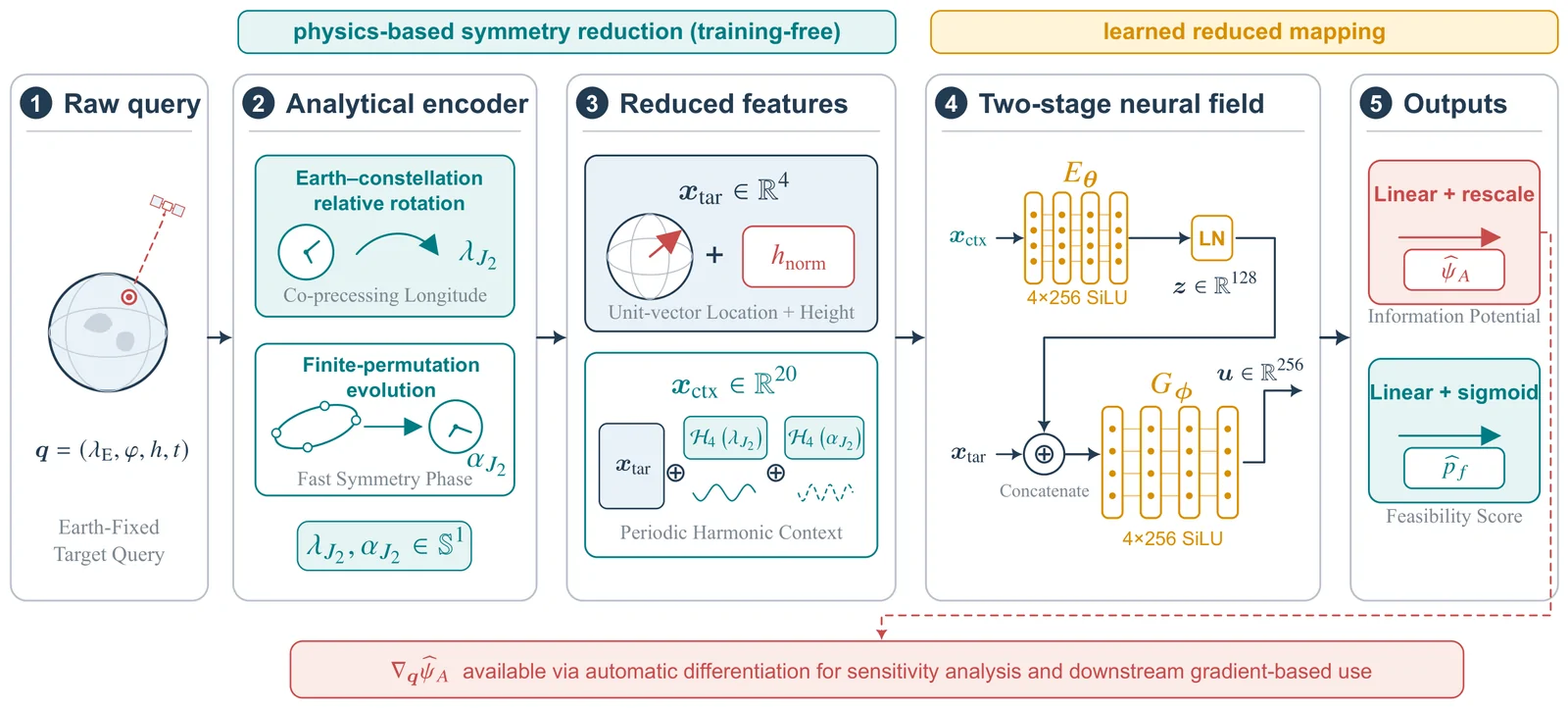
Symmetry-reduced neural information-field architecture.

Figure 6 shows how the analytical reduction and the learned mapping form the complete neural information field.

The parameter set P fixes $t_{0}$, $\theta_{G,0}$, $\omega_{E}$, $\Omega_{0}$, $M_{0}$, $K_{\mathrm{sym}}$, $\Delta \Omega_{\Pi }$, $T_{\mathrm{rp}}$, ${\dot{\Omega }}_{\mathrm{rp}}$, the altitude interval, the frame convention, and the sensing model. These quantities determine the physical field but are not optimized by $f_{\Theta }$. Consequently, application to another epoch convention, Walker geometry, or sensing configuration requires either a consistent transformation into the same parameterization or a separately trained field.

### 3.2. Reduced Coordinate Features

The target branch receives a continuous embedding of location and altitude. Define

(27) $$h_{\mathrm{norm}}=\frac{h-h_{\min}}{h_{\max}-h_{\min}},$$

and

(28) $$x_{\mathrm{tar}}=\left[\begin{array}{c} \cos\varphi \cos\lambda_{J_{2}}\\ \cos\varphi \sin\lambda_{J_{2}}\\ \sin\varphi \\ h_{\mathrm{norm}} \end{array}\right]\in R^{4}.$$

The first three entries form a unit-vector embedding in the co-precessing frame. This representation maps equivalent angular endpoints to the same feature vector, while the fourth entry preserves altitude as a continuous interpolation coordinate.

To represent phase-dependent spatial structure at several frequencies, let

(29) $$H_{L}\left(\beta \right)={\left[\begin{array}{ccccc} \sin\beta & \cos\beta & \cdots & \sin(L\beta ) & \cos(L\beta ) \end{array}\right]}^{T}.$$

The reduced phase-context feature is

(30) $$x_{\mathrm{ctx}}=\left[\begin{array}{c} x_{\mathrm{tar}}\\ H_{L}\left(\lambda_{J_{2}}\right)\\ H_{L}\left(\alpha_{J_{2}}\right) \end{array}\right]\in R^{4+4L}.$$

Harmonic coordinate features allow a multilayer perceptron to represent periodic high-frequency variation without receiving a discontinuous wrapped angle [19]. The adopted basis uses the first four harmonics (L=4), giving a 20-dimensional context input and a four-dimensional target input.

The two occurrences of $\lambda_{J_{2}}$ in Equations (28) and (30) have distinct roles. The unit-vector terms provide a continuous spherical representation of target position, including its latitude dependence. The terms in $H_{L}\left(\lambda_{J_{2}}\right)$ provide a multiscale basis for variation relative to the rotating constellation geometry, and the harmonics of $\alpha_{J_{2}}$ resolve changes associated with the finite satellite permutation. Because Equation (30) also includes $x_{\mathrm{tar}}$, the resulting latent vector remains a target-conditioned phase-context descriptor rather than a target-independent constellation state.

### 3.3. Two-Stage Neural Architecture

The first stage maps the reduced context to a normalized latent vector,

(31) $$z=\mathrm{LN}\left[E_{\theta }\left(x_{\mathrm{ctx}}\right)\right]\in R^{128},$$

where $E_{\theta }$ is a multilayer perceptron and LN denotes layer normalization. The second stage combines this descriptor with the explicit target features,

(32) $$u=G_{\phi }\left(\left[\begin{array}{c} x_{\mathrm{tar}}\\ z \end{array}\right]\right)\in R^{256},$$

which is passed through two scalar branches and their output transformations,

(33) $$\begin{array}{cc} {\hat{y}}_{\psi }=a_{\psi }^{T}u+b_{\psi },\; & {\hat{\eta }}_{f}=a_{f}^{T}u+b_{f},\\ {\hat{\psi }}_{A}=s_{\psi }{\hat{y}}_{\psi }+\mu_{\psi },\; & {\hat{p}}_{f}=\frac{1}{1+\exp(-{\hat{\eta }}_{f})}, \end{array}$$

where $\mu_{\psi }$ and $s_{\psi }$ are fitted only from feasible training labels.

The backbone receives target geometry explicitly through $x_{\mathrm{tar}}$ and through its phase-conditioned descriptor z. The potential and feasibility branches share this representation because both outputs depend on the same visibility and geometry conditions.

Table 1 summarizes the architecture. All hidden transformations use the smooth sigmoid linear unit (SiLU) activation. The information backbone includes a final SiLU activation before the two linear output branches.

The complete model contains 533,634 trainable parameters.

### 3.4. Training Targets and Objective

For sample *n*, let $f_{n}\in \left\{0,1\right\}$ be the feasibility label defined in Section 2.2. Feasible potential labels are standardized using training-set statistics,

(34) $$y_{n}=\frac{\psi_{A,n}-\mu_{\psi }}{s_{\psi }},\;f_{n}=1.$$

The potential-output branch is trained only on feasible samples. With $e_{n}={\hat{y}}_{\psi ,n}-y_{n}$, the unit-transition Huber penalty is

(35) $$H_{1}\left(e\right)=\left\{\begin{array}{cc} \frac{1}{2}e^{2}, & |e|<1,\\ \left|e\right|-\frac{1}{2}, & |e|\geq 1. \end{array}\right.$$

The affine standardization in Equation (34) sets the scale of the regression problem without changing the physical interpretation of the output. The Huber penalty is quadratic for |e|<1 and linear outside that interval, thereby limiting the influence of large residuals wherever they occur. It does not by itself determine the behavior of the approximation near visibility-set or optimal-subset transitions. The potential and feasibility losses are

(36) $$L_{\psi }=\frac{\sum_{n=1}^{B}f_{n}H_{1}\left(e_{n}\right)}{\max\left(1,\sum_{n=1}^{B}f_{n}\right)},$$

and

(37) $$L_{f}=-\frac{1}{B}\sum_{n=1}^{B}\left[f_{n}\log{\hat{p}}_{f,n}+\left(1-f_{n}\right)\log\left(1-{\hat{p}}_{f,n}\right)\right],$$

where *B* is the minibatch size. The total objective is

(38) $$L=L_{\psi }+\beta_{f}L_{f},\;\beta_{f}=0.1.$$

All feasible samples contribute equally to $L_{\psi }$, and the latent descriptor is learned through the joint potential and feasibility objectives without additional latent supervision. The feasibility branch identifies the domain on which the selected-subset potential is defined. Its score is thresholded at 0.5 for this purpose and is not interpreted as a calibrated probability.

### 3.5. Differentiability and Scope of Use

The analytical encoding and neural network form a differentiable potential-prediction path. Sine and cosine phase coordinates, linear altitude normalization, SiLU activations, and layer normalization admit derivatives with respect to longitude, latitude, altitude, and time within the continuous coordinate chart. For example,

(39) $$\nabla_{q}{\hat{\psi }}_{A}={\left[\begin{array}{cccc} \partial {\hat{\psi }}_{A}/\partial \lambda_{E} & \partial {\hat{\psi }}_{A}/\partial \varphi & \partial {\hat{\psi }}_{A}/\partial h & \partial {\hat{\psi }}_{A}/\partial t \end{array}\right]}^{T}$$

can be obtained without finite-differencing the orbit propagator and subset search. The spherical longitude coordinate remains degenerate at the poles, so Cartesian directional derivatives or a local chart are preferable there.

At fixed Earth-fixed target coordinates, the temporal derivative separates into the relative rotation of the Earth and the unlabeled constellation pattern and the fast finite-permutation evolution:

(40) $$\frac{\partial {\hat{\psi }}_{A}}{\partial t}=\left(\omega_{E}-{\dot{\gamma }}_{\mathrm{sym}}\right)\frac{\partial {\hat{\psi }}_{A}}{\partial \lambda_{J_{2}}}+\frac{2\pi K_{\mathrm{sym}}}{T_{\mathrm{rp}}}\frac{\partial {\hat{\psi }}_{A}}{\partial \alpha_{J_{2}}}.$$

Equation (40) makes the physical origin of time sensitivity explicit while retaining the bounded phase representation.

The reference potential can develop jumps when changes in visibility or candidate-pool membership alter the admissible subsets. With an unchanged candidate pool, crossings between smooth subset potentials can instead produce continuous but nondifferentiable minima. Neural gradients describe sensitivities of the smooth approximation; gradient agreement away from transitions and value errors near transitions are therefore evaluated separately.

Differentiable predictions are restricted to the fixed parameter set P and the sampled spatial domain. The $J_{2}$ phases remove absolute-time growth for the relative-periodic model, but additional forces and parameter differences can violate the relative-periodic relation or alter its characteristic rates, causing the reduced field to diverge gradually from higher-fidelity dynamics. These deviations determine the validity horizon quantified in Section 4.

## 4. Experiment

The experiments separate three error sources: numerical symmetry closure, neural approximation on the reduced domain, and dynamics mismatch during field reuse. Parameter, topology, application, and timing tests then define the conditions under which the field remains useful.

### 4.1. Common Experimental Configuration and Evaluation Protocol

Unless otherwise stated, the reference case was a 25×25 Walker Delta constellation at a satellite orbital altitude of 500 km, with F=1 and $i=86^{\circ }$. The numerical $J_{2}$ orbit had $T_{\mathrm{rp}}=5676.9223$ s, $\Delta t_{\mathrm{slot}}=227.0769$ s, and ${\dot{\Omega }}_{\mathrm{rp}}=-1.08136\times 10^{-7}$ rad/s. Each satellite supplied two angular components, each with a standard deviation of 10 arcsec. Availability required a range below 2800 km and Earth clearance above 5 km; field-of-view constraints were excluded. All experiments set $N_{\mathrm{sel}}=3$; the candidate-pool reference searched every triplet among the 12 largest individual range weights using the unregularized FIM and a reciprocal-condition-number threshold of $10^{-11}$. Table 2 lists the remaining fixed settings.

Training used $H_{\mathrm{tr}}=\left\{15,25,\ldots ,95,100\right\}$ km and 64 uniformly spaced values of the fast phase $\alpha_{J_{2}}$ over one symmetry period. At each phase, the $1^{\circ }$ longitude–latitude grid spans the full co-precessing longitude $\lambda_{J_{2}}$; the periodic endpoint was retained only for closure supervision and diagnostics. Validation used $H_{\mathrm{val}}=\left\{20,30,\ldots ,90,97.5\right\}$ km at all 64 half-step phase offsets, and testing used $H_{\mathrm{te}}=\left\{17.5,22.5,\ldots ,92.5,96.25,98.75\right\}$ km at 32 quarter- and three-quarter-step phases. Training and validation used latitude-stratified samples of 10,000,000 and 2,000,000 points, respectively; testing evaluated all 37,636,416 grid points. Test samples were unseen in both altitude and phase.

The matched two-body encoding (TB) omits nodal precession and uses

(41) $$\lambda_{\mathrm{TB}}\equiv \lambda_{E}+\theta_{G,0}+\omega_{E}\Delta t-\Omega_{0}\;\left(\mathrm{mod}\;2\pi \right),\;\alpha_{\mathrm{TB}}\equiv N_{s}\left(\frac{2\pi \Delta t}{T_{\mathrm{TB}}}+M_{0}\right)\;\left(\mathrm{mod}\;2\pi \right),$$

where $T_{\mathrm{TB}}=5676.9780$ s is the two-body period. Its feature vectors follow Equations (28)–(30) with $\left(\lambda_{J_{2}},\alpha_{J_{2}}\right)$ replaced by $\left(\lambda_{\mathrm{TB}},\alpha_{\mathrm{TB}}\right)$. TB and the proposed $J_{2}$ symmetry-reduced encoding (J2-SR) used the same numerical-$J_{2}$ labels, sample indices, architecture, optimization, model-selection rule, and three random seeds; only the coordinate representation differed. The paired RMSE reduction was quantified by 10,000 bootstrap resamples of 576 matched altitude–phase slices. The 2.5th and 97.5th percentiles define the reported 95% interval. Training seeds were held fixed in this calculation and their variability was reported separately.

Field accuracy was assessed by root-mean-square error (RMSE), mean absolute error (MAE), $R^{2}$, error quantiles, and altitude- and phase-resolved errors. The poor-geometry subset comprises the upper 20% of reference $\psi_{A}$ values. Feasibility scores were thresholded at 0.5 and evaluated by accuracy and, when both classes occurred, the F1 score. Altitude gradients were compared with centered finite differences using RMSE, Pearson correlation, and sign agreement; value and gradient closure were checked at periodic endpoints. Orbital-model tests evaluated state closure, energy conservation, and field symmetry. For vector residuals $e_{i}$, RMS denotes $[N^{-1}\sum_{i}∥e_{i}{{∥}_{2}^{2}]}^{1/2}$, whereas RMSE refers to scalar field differences. For an external field $\psi_{A}^{\mathrm{ext}}\left(t\right)$ and neural prediction ${\hat{\psi }}_{A}\left(t\right)$, the validity horizon is

(42) $$T_{\mathrm{valid}}=\inf\left\{t_{k}:\left({\mathrm{RMSE}}_{\psi }\left(t_{j}\right)>0.005\vee R_{\psi }^{2}\left(t_{j}\right)<0.99\right),\;j=k,k+1,k+2\right\},$$

on the regular 6 h grid. The validity horizon is the first time at which three consecutive samples exceed either accuracy threshold. If no such sequence occurs, the horizon is reported as at least the final evaluation time. The primary thresholds, RMSE =0.005 and $R^{2}=0.99$, were fixed before external propagation as engineering tolerances for field reuse, independent of filtering-error thresholds. Because $\psi_{A}$ is logarithmic, a potential difference of 0.005 corresponds to a factor $10^{0.005}=1.0116$ in the A-optimal uncertainty scale. RMSE thresholds of 0.003 and 0.010 provide sensitivity checks.

Higher-fidelity tests were conducted with TudatPy 1.0.0 [30] at three absolute epochs and target altitudes of 20, 50, and 97.5 km, using approximately 20,000 latitude-balanced samples per time. Matched-$J_{2}$ and high-fidelity runs used the same 625 Earth-centered J2000 initial states and output epochs. The high-fidelity force model combined the static GOCO05c gravity field to degree and order 70 [31], solar and lunar point-mass gravity, cannonball SRP with Earth occultation, and NRLMSISE-00 drag [32]. Each satellite had a mass of 100 kg, drag and SRP areas of 1 m^2^, $C_{D}=2.2$, and $C_{R}=1.3$. The atmosphere co-rotated with Earth and used an archived CelesTrak historical space-weather file with storm indices and anomalous oxygen enabled. Runtime medians and interquartile ranges (IQRs) were calculated from 30 timed repetitions following 20 untimed stabilization runs.

### 4.2. Relative-Periodic $J_{2}$ Model and Symmetry Validation

Bounded-phase validity requires the propagated $J_{2}$ Walker constellation to satisfy the return relation defined by the rotation and permutation. Table 3 reports the resulting adjacent-slot and full-period residuals.

**Figure 7:**
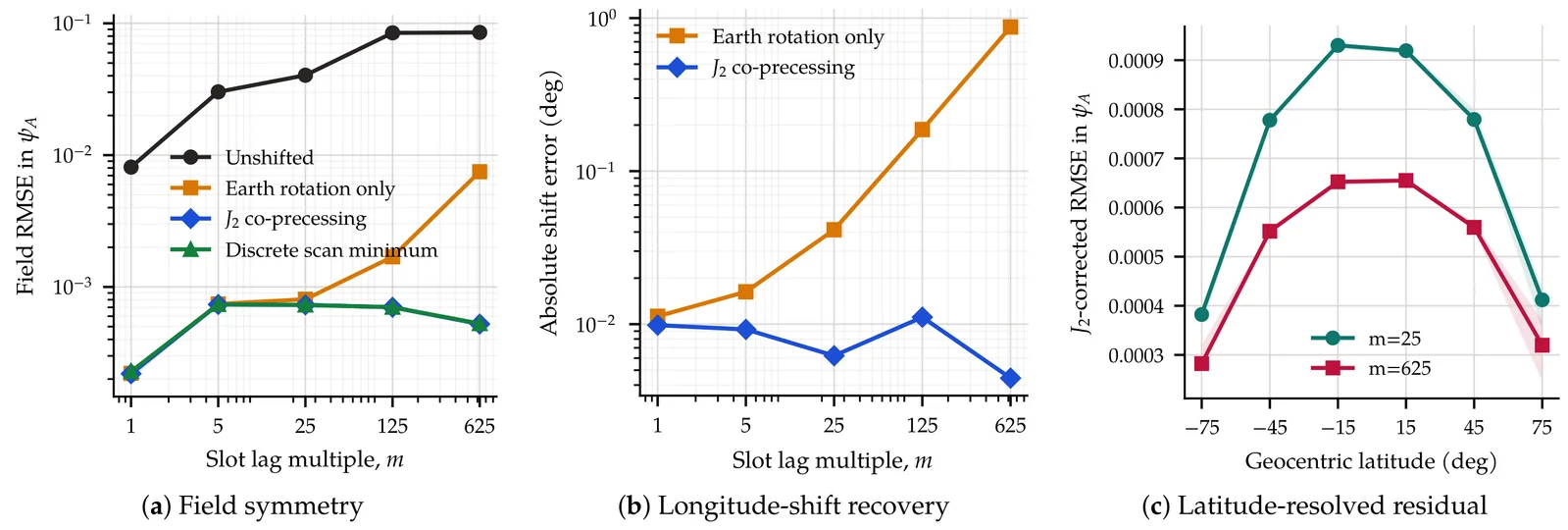
Numerical validation of the $J_{2}$ symmetry reduction.

The state residuals were below $10^{-9}$ km in position and $10^{-12}$ km/s in velocity. Field symmetry was then tested at $\Delta t=m\tau_{\mathrm{sym}}$, m∈{1,5,25,125,625}, over three base epochs. Figure 7 compares no alignment, Earth rotation, the analytical $J_{2}$ shift, and an independent longitude scan. Curves report the median over the three epochs, and bands span their range. The $J_{2}$-aligned RMSE remained below $7.37\times 10^{-4}$ through 39.4 h; at m=625 it was $5.23\times 10^{-4}$, compared with $7.49\times 10^{-3}$ for Earth rotation and $8.53\times 10^{-2}$ without alignment.

At m=625, the analytical shift differed from the scan optimum by $0.00444^{\circ }$, compared with $0.875^{\circ }$ for Earth rotation alone, and all latitude-band RMSE values remained below $9.39\times 10^{-4}$. Together with the state-closure residuals, this agreement validates the analytical $J_{2}$ shift and bounded-phase construction over 39.4 h independently of neural approximation.

### 4.3. Neural-Field Accuracy, Interpolation, and Differentiability

Table 4 compares TB and J2-SR on jointly unseen altitude–phase samples. J2-SR yielded lower RMSE for all three seeds, reducing the mean RMSE from $7.664\times 10^{-3}$ to $1.154\times 10^{-3}$; its worst-seed RMSE was $1.445\times 10^{-3}$.

**Figure 8:**
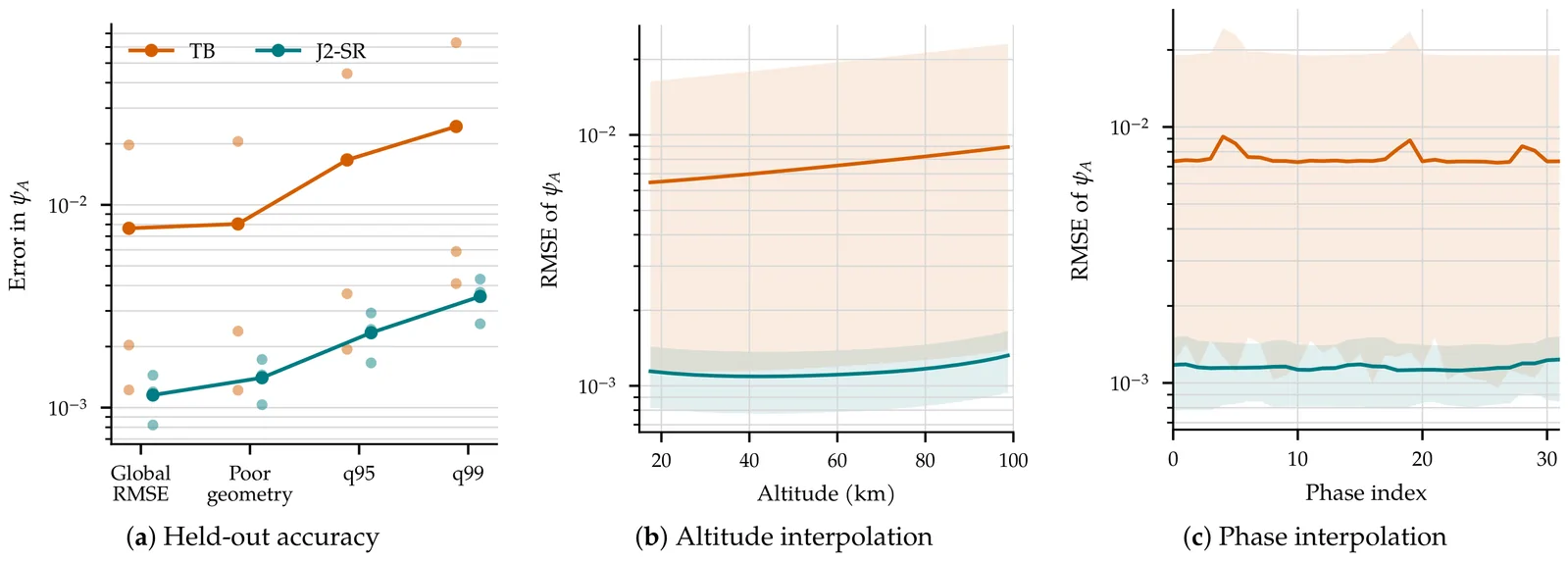
Representation ablation and interpolation accuracy.

The arithmetic means of the three seed-wise RMSE values give an 84.94% reduction. The predefined paired analysis instead pooled weighted squared errors over 576 matched altitude–phase slices and the three fixed seed pairs before taking the RMSE ratio; this estimand gave a reduction of 89.70%, with a 95% paired bootstrap interval of 89.61–89.79%. The interval quantifies variation across matched slices conditional on the three trained seed pairs and does not estimate population-level training uncertainty. Figure 8 shows the individual seeds and the resolved interpolation errors. Pale markers denote individual seeds; solid curves denote seed means, and bands span the seed extrema.

The J2-SR altitude-resolved RMSE ranged from $1.090\times 10^{-3}$ to $1.321\times 10^{-3}$ over 17.5–98.75 km, and its phase-resolved RMSE ranged from $1.119\times 10^{-3}$ to $1.234\times 10^{-3}$. These ranges characterize interpolation within the stated domain. A separate seed-2026 capacity check gave RMSE values of 0.003071, 0.001445, and 0.0004959 for networks with 268,434, 533,634, and 810,306 parameters, respectively. The 533,634-parameter architecture used throughout was the intermediate-capacity case. Holding this capacity equal in TB and J2-SR isolates the coordinate representation in the primary comparison.

**Figure 9:**
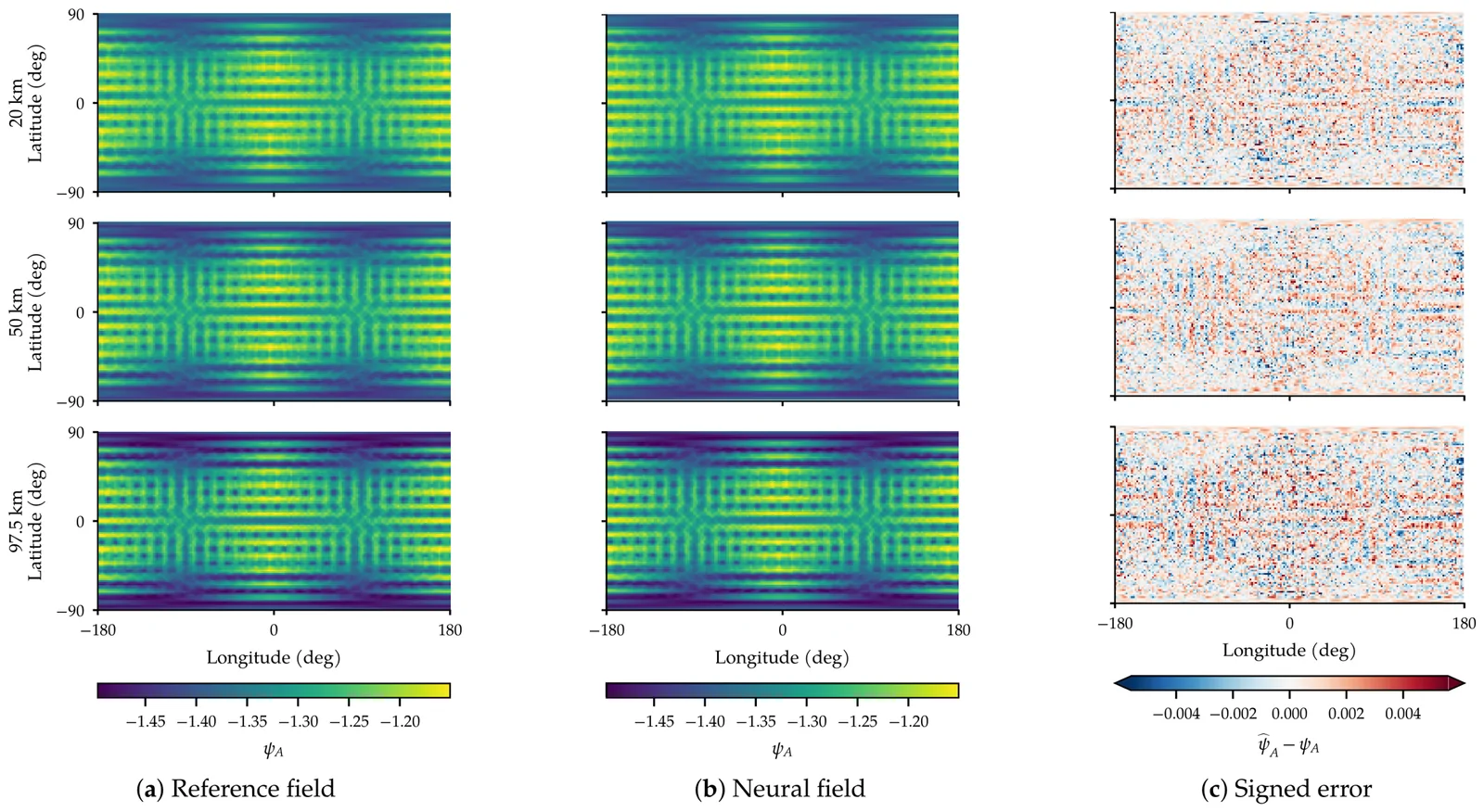
Joint information-field interpolation at three altitudes.

Figure 9 compares reference and neural fields at an unseen phase. At 20, 50, and 97.5 km, RMSE was $1.473\times 10^{-3}$, $1.449\times 10^{-3}$, and $1.715\times 10^{-3}$, respectively, with $R^{2}\geq 0.99952$. For visualization only, the signed-error color scale is clipped at the 99.5th percentile of the absolute error; all samples, including those outside the displayed color range, were retained in the RMSE, MAE, $R^{2}$, and all other reported metrics.

**Figure 10:**
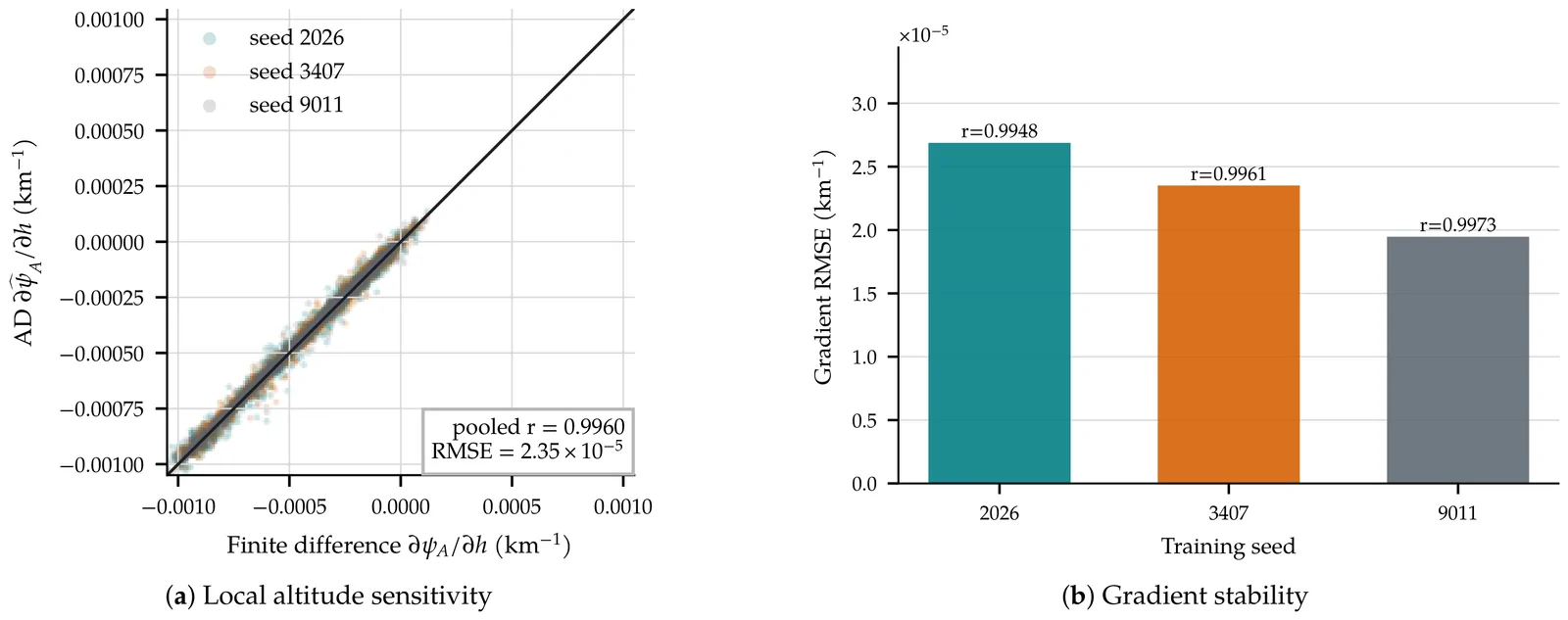
Local altitude-gradient agreement and stability across training seeds.

Figure 10 compares neural automatic-differentiation gradients with centered finite differences. The comparison used up to 10,000 feasible samples per seed at 52.5 km whose absolute reference second difference across 47.5, 52.5, and 57.5 km did not exceed its 90th percentile. This criterion excludes regions near sharp altitude transitions, where the discrete reference may be nonsmooth.

Across 30,000 samples, automatic-differentiation and finite-difference altitude gradients had a Pearson correlation of 0.9960, an RMSE of $2.35\times 10^{-5}$ km^−1^, and 99.17–99.42% sign agreement across seeds. Periodic value closure was exact to machine precision, and gradient-closure RMSE was below $3.80\times 10^{-16}$.

**Figure 11:**
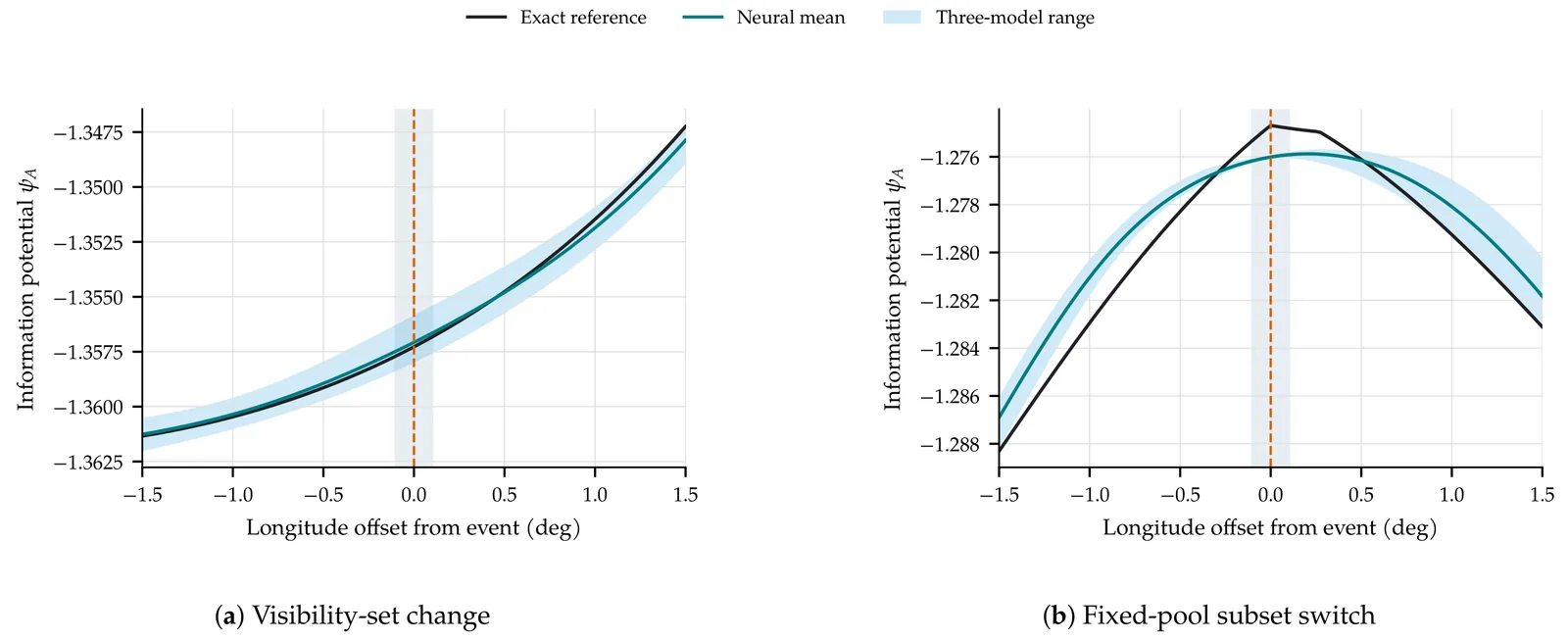
Neural approximation near geometric transitions. The candidate pool is unchanged across both marked events. The vertical dashed line locates the event, and the shaded strip marks the $\pm 0.1^{\circ }$ boundary band.

Visibility changes and optimal-subset switches have different effects on field regularity. Across 72 longitude scans spanning three target altitudes, three latitudes, and eight phases, none of the 24,484 localized visibility crossings changed the 12-satellite candidate pool. At the 30 selected visibility events, the optimal subset also remained unchanged. In contrast, all 30 selected subset switches occurred within unchanged visible sets and candidate pools. Independent evaluation of the competing subset potentials confirmed continuous crossings with unequal one-sided slopes, identifying kinks in the reference potential. Figure 11 illustrates this distinction.

Table 5 compares the errors of three fixed models near these events. Each profile used a uniform $0.025^{\circ }$ longitude spacing over $\pm 1.5^{\circ }$ from the localized event. The boundary band covered $\pm 0.1^{\circ }$; the off-event neighborhood covered distances of 0.5–$1.5^{\circ }$ and could contain other transitions. Event selection and localization used only the exact reference; complete event definitions, one-sided convergence checks, and per-event statistics are provided in the accompanying data.

The mean boundary-band RMSE was 0.001335 for visibility-set changes and 0.002479 for subset switches. The larger error near subset switches than in their off-event neighborhoods reflects the greater approximation difficulty at these kinks. Although every model’s RMSE pooled across each event class remained below 0.005, seven of the 180 event–model pairs exceeded this value individually, with a maximum local RMSE of 0.00660. Thus, the pooled accuracy does not ensure uniformly small local errors. All sampled profiles remained feasible; visibility-induced potential jumps and feasible–infeasible transitions were not covered.

### 4.4. Applicability and Sensitivity Boundaries

The sensitivity analysis examines temporal consistency under matched dynamics, the effects of constellation parameters and topology at fixed network capacity, and the tolerance of trained fields to differential orbital-element drift.

#### 4.4.1. Long-Horizon Consistency Under Matched $J_{2}$ Dynamics

Long-horizon consistency was assessed over 30 days using the same $J_{2}$ dynamics for field construction and evaluation. On a global $2^{\circ }$ grid at 14 times, the overall RMSE was $1.512\times 10^{-3}$, the maximum time-resolved RMSE was $1.581\times 10^{-3}$, and the minimum $R^{2}$ was 0.999578 over 691,782 feasible samples. Targeted $1^{\circ }$ evaluations at days 0, 7, and 30 remained below $1.695\times 10^{-3}$ with $R^{2}\geq 0.999532$. The sampled reference-closure error of $3.357\times 10^{-3}$ is attributable to grid discretization because the $2^{\circ }$ grid cannot represent the $0.95015^{\circ }$ one-slot shift exactly. The 30-day result confirms consistency under matched $J_{2}$ dynamics. Exact neural endpoint closure follows algebraically from the periodic representation and was excluded from external physical validation.

#### 4.4.2. Constellation Parameter Sensitivity

The analysis considered nine one-factor-at-a-time configurations that varied constellation scale (20×20–30×30), inclination (70–$86^{\circ }$), satellite orbital altitude (400–600 km), and phasing (F=0,1,12). Each case used an independently generated candidate-pool reference field and three independently trained models; architecture and training budget were fixed.

**Figure 12:**
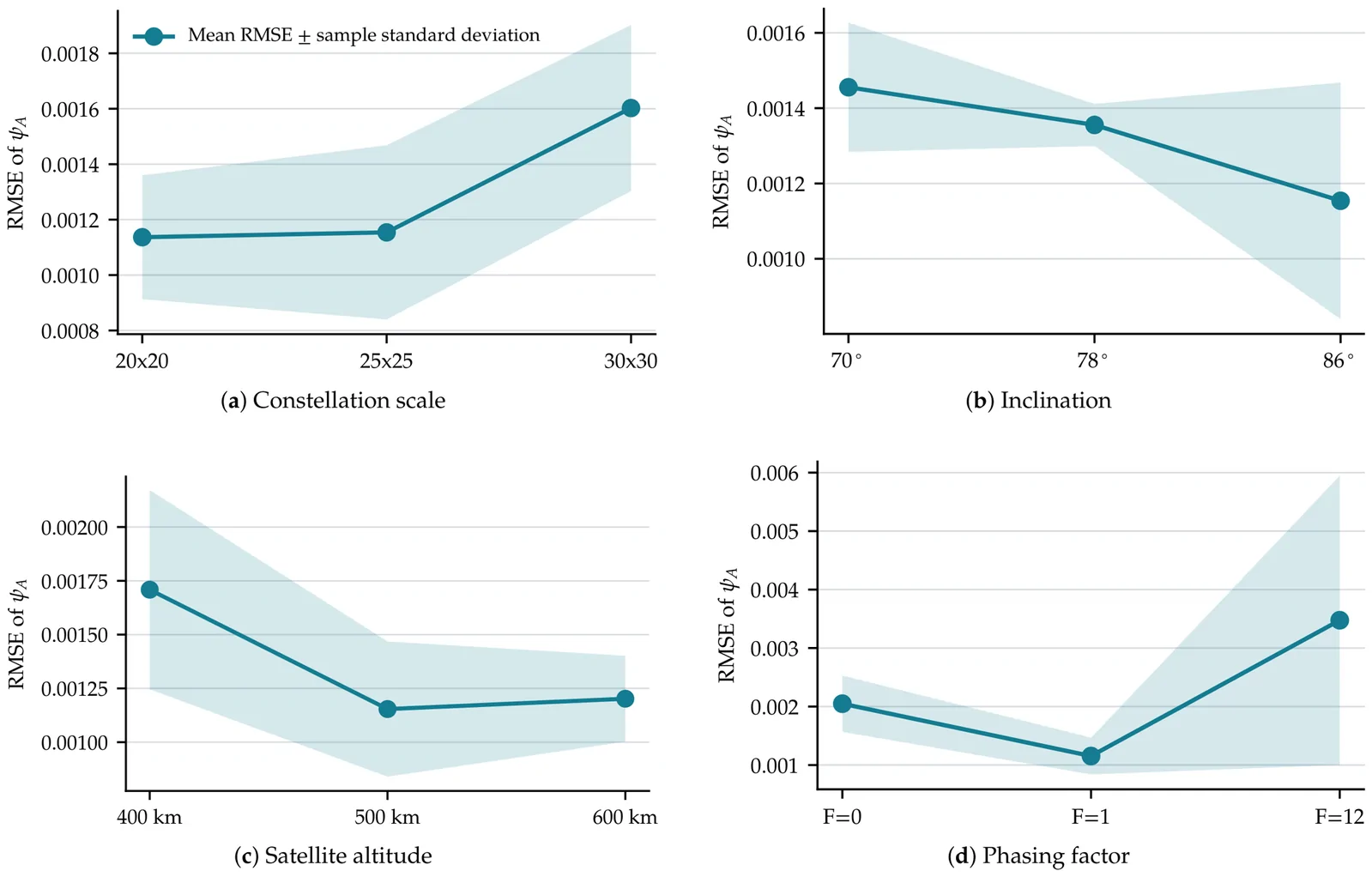
Sensitivity to constellation parameters under a fixed neural architecture.

Mean RMSE across the nine configurations ranged from $1.137\times 10^{-3}$ to $3.476\times 10^{-3}$ (Figure 12 and Table 6). Curves report the three-seed mean, and bands report one sample standard deviation. Scale, inclination, and satellite-altitude cases remained below $1.709\times 10^{-3}$. The greatest sensitivity occurred at F=12, with a mean RMSE of $3.476\times 10^{-3}$, a worst-seed RMSE of $6.275\times 10^{-3}$, and a worst altitude–phase group RMSE of 0.0261. Symmetry reduction remained valid across all tested settings, whereas errors for some seeds and local groups exceeded 0.005 with the fixed architecture. Network capacity should therefore be reassessed after a change in constellation parameters.

#### 4.4.3. Applicability to Delta, Star, and Rosette Constellation Topologies

**Figure 13:**
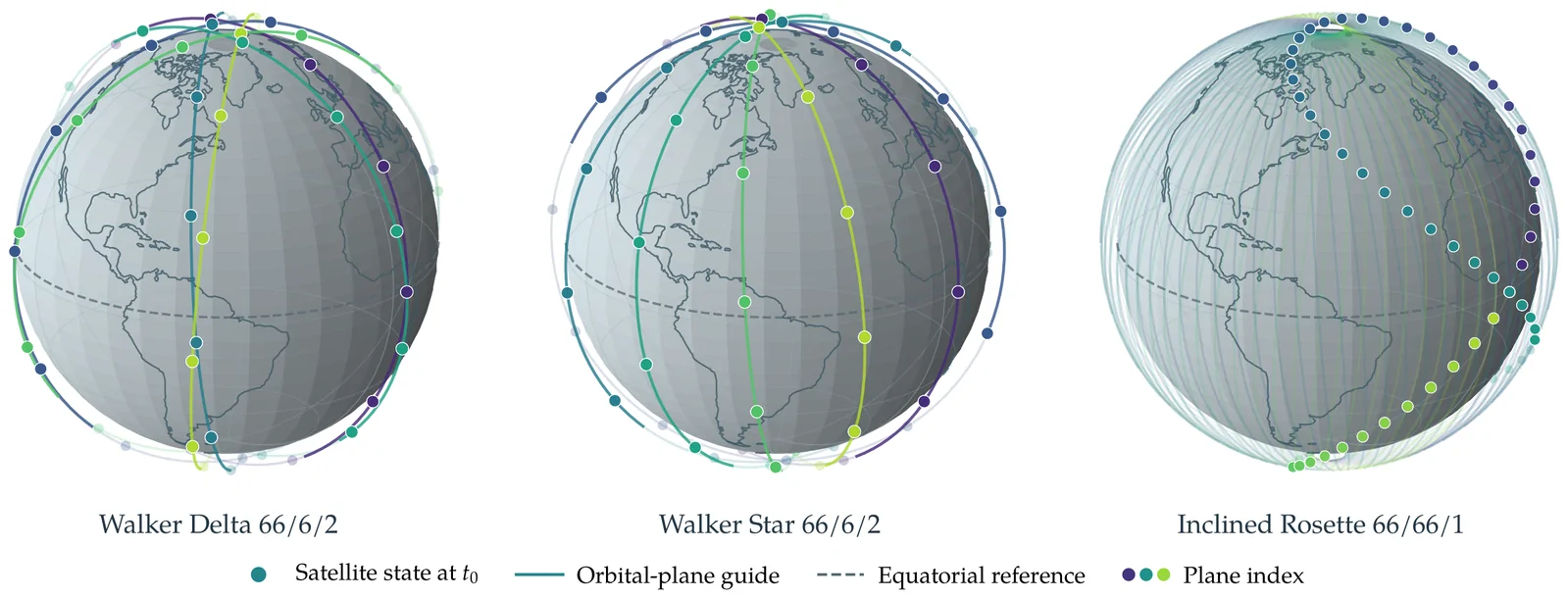
Common-epoch geometry of the three constellation topologies.

The topology test considered three 66-satellite configurations at a satellite orbital altitude of 500 km and an inclination of $86^{\circ }$: Walker Delta 66/6/2, Walker Star 66/6/2, and inclined Rosette 66/66/1. Figure 13 shows their common-epoch geometry. Delta and Star each contain six planes with 11 satellites per plane; their nodes are uniformly distributed over $360^{\circ }$ and $180^{\circ }$ in RAAN, respectively. The inclined Rosette assigns one satellite to each of 66 planes, with nodes distributed over $360^{\circ }$.

The satellite markers show positions from the $J_{2}$-consistent initial states used in the topology experiment. The curves indicate orbital-plane geometry rather than propagated trajectories, and the coastlines provide orientation under the adopted $\theta_{G,0}=0$ convention. The closure analysis used in-plane cyclic permutations of order 11 for Delta and Star and a cross-plane cyclic permutation of order 66 for Rosette. Feasibility disagreement is the fraction of queries for which the mapped and recomputed indicators differ. For a base optimum $C^{★}$, mapped-subset regret is $\max[0,\psi_{A}\left(\Pi^{-1}C^{★}\right)-\psi_{A}\left(C_{\mathrm{new}}^{★}\right)]$ after the symmetry transformation.

**Figure 14:**
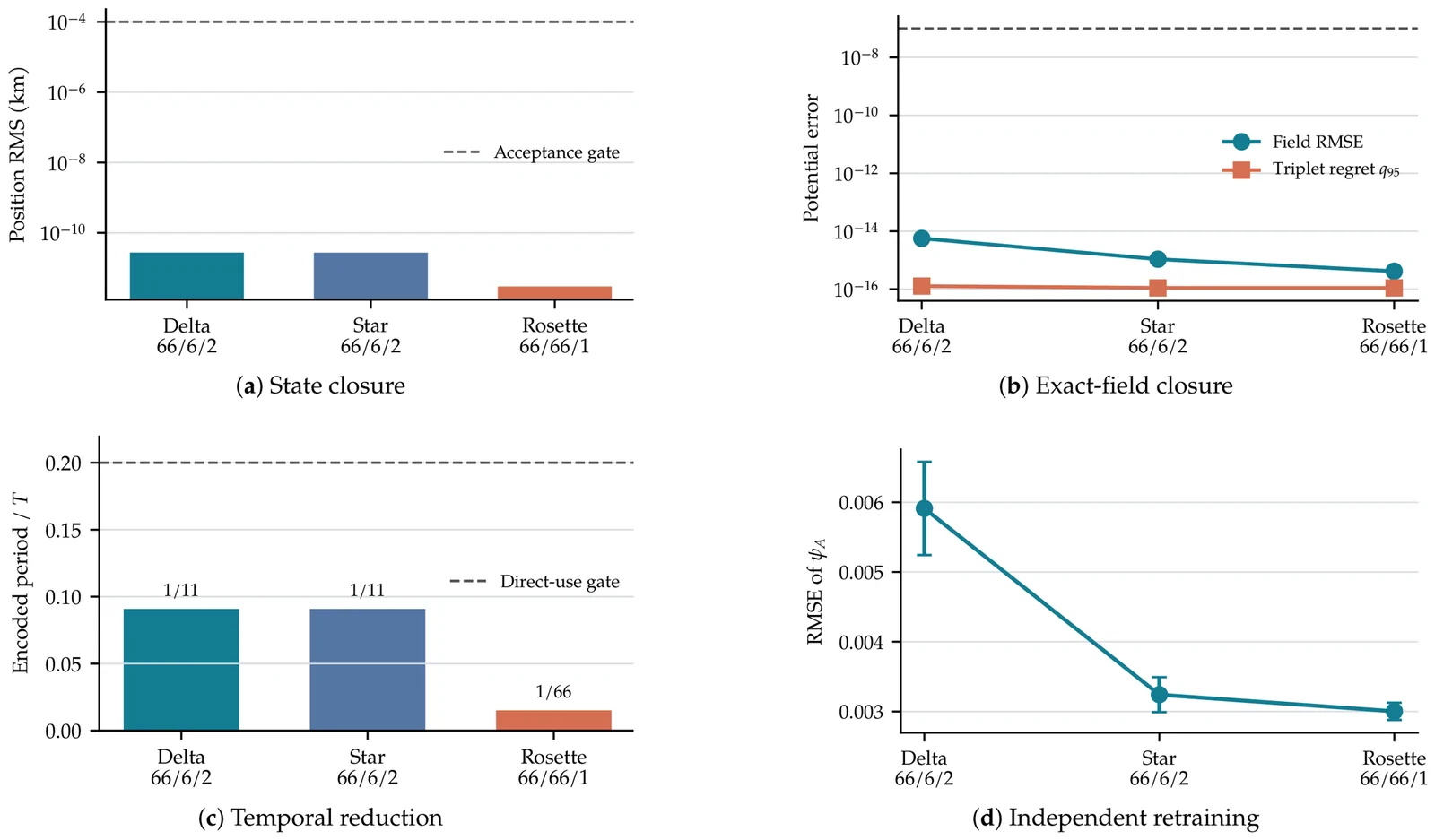
Finite-symmetry closure and neural accuracy across three constellation topologies.

All three cases achieved numerical state and reference-field closure, with RMS position-closure residuals below $2.76\times 10^{-11}$ km, field RMSE below $5.67\times 10^{-15}$, zero feasibility disagreement, and mapped-subset regret at the $10^{-16}$ level. Figure 14 shows mean RMSE values of $5.912\times 10^{-3}$, $3.241\times 10^{-3}$, and $3.003\times 10^{-3}$ for independently retrained Delta, Star, and Rosette fields, respectively. Their worst-seed feasibility F1 scores were 0.9958, 0.9925, and 0.9996. The Delta error exceeded the 0.005 accuracy threshold; the Star and Rosette errors remained below it. Delta also satisfied the state- and exact-field-closure tests at numerical precision, so its larger neural error does not indicate failure of the finite symmetry relation. The result instead shows greater approximation difficulty for the Delta mapping under the common 533,634-parameter architecture and fixed training budget. Possible contributors include topology-dependent local field variation, visibility and best-subset transition structure, and finite model or optimization capacity. These factors were not varied independently and cannot be assigned a unique causal contribution from the present comparison. In the denser 25×25 reference case, every test query was feasible and all were classified as feasible, giving an accuracy of 1.000; precision and recall are not separately informative for that single-class test set.

#### 4.4.4. Fixed-Field Tolerance to Differential Orbital-Element Drift

To quantify local robustness before a discrete constellation change, zero-mean differential perturbations were applied separately to semi-major axis *a*, inclination *i*, right ascension of the ascending node Ω, and mean argument of latitude $u_{m}=\omega +M$. For satellite *j*, each perturbation had the form $\delta x_{j}=A_{x}q_{j}$, where $A_{x}$ is the prescribed satellite-wise RMS amplitude and $q_{j}$ is a fixed zero-mean, unit-RMS coefficient. Satellite-wise independent, plane-correlated, and slot-correlated patterns were held fixed across amplitudes and propagated under $J_{2}$. The three fields were evaluated without retraining at eight phases within one nominal slot-repeat period and three target altitudes, using 512 common locations per phase–altitude pair. Accuracy was assessed over all patterns, training seeds, phases, and altitudes, requiring a maximum total RMSE of at most 0.005 and a minimum $R^{2}$ of 0.99.

**Figure 15:**
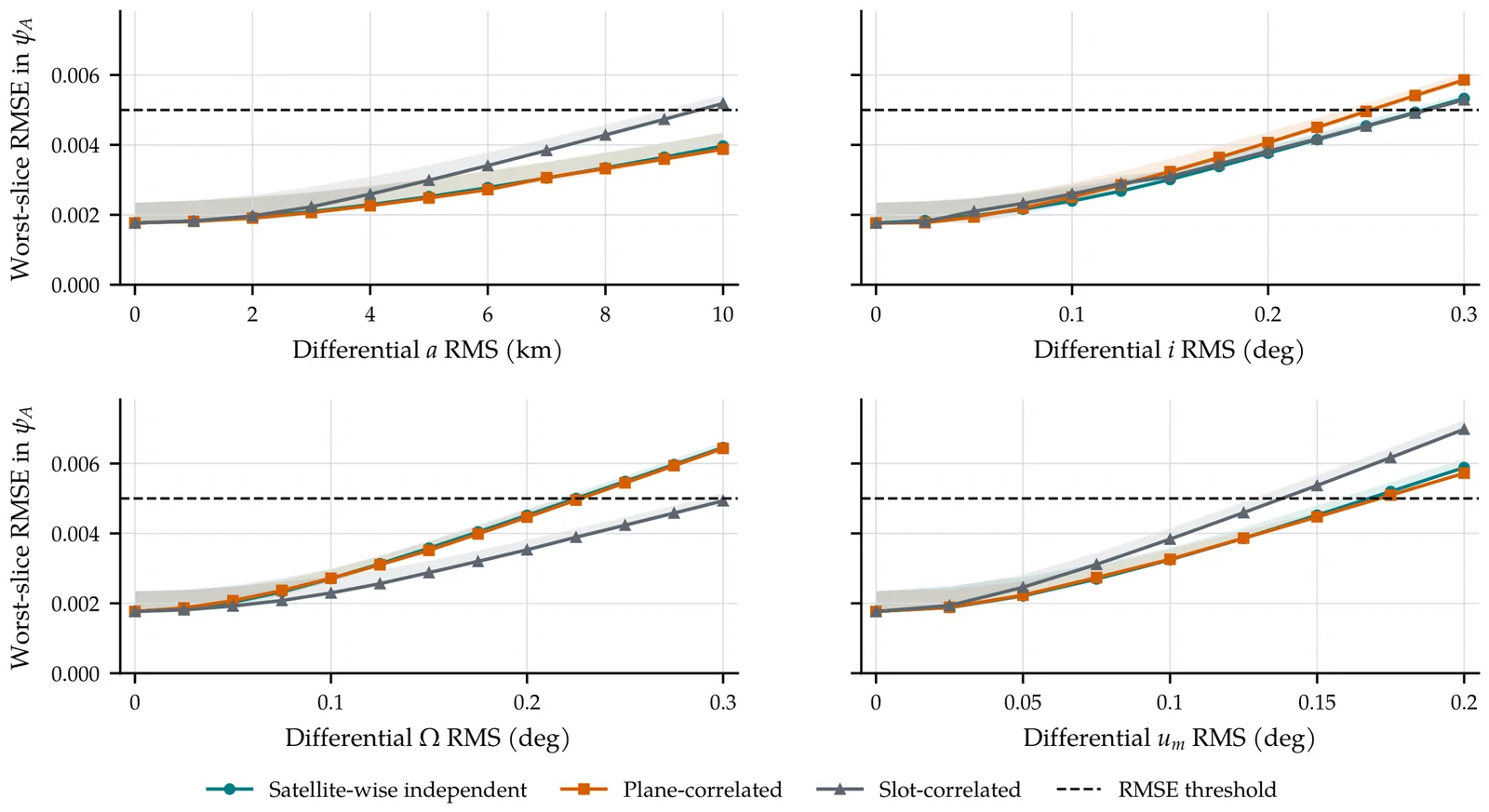
Sensitivity of fixed neural fields to differential orbital-element drift.

For each perturbation pattern and amplitude, Figure 15 shows the median across the three fields after taking each field’s worst phase–altitude RMSE; the bands span the corresponding minimum and maximum. Starting from a zero-drift maximum RMSE of 0.002348, each orbital-element sweep crossed the 0.005 threshold within the sampled range. In every case, $R^{2}$ remained above 0.99, so RMSE determined the tolerance limit. Table 7 brackets each transition between adjacent sampled RMS amplitudes; these intervals locate threshold crossings without implying an exact continuous boundary.

These sampled intervals are configuration-specific and characterize one-element-at-a-time robustness. They do not constitute a joint validity guarantee when several orbital elements drift simultaneously.

### 4.5. High-Fidelity Analysis

A matched-$J_{2}$ cross-propagator comparison isolates numerical implementation error. Uncontrolled high-fidelity propagation and historical Iridium trajectories then assess the validity horizon for a simulation without orbit maintenance and for historical operational trajectories.

#### 4.5.1. Matched-$J_{2}$ Cross-Propagator Consistency

The consistency test propagated the same 625 Earth-centered J2000 states with the reference model and TudatPy under matched central gravity and $J_{2}$, at three epochs and four elapsed times through 6 h. Both histories were transformed independently and evaluated as candidate-pool reference fields.

The first three rows of Table 8 summarize the 30-day neural-field results under the reference $J_{2}$ dynamics; all remained within the field-accuracy thresholds. The final five rows isolate the 6 h cross-propagator comparison. Its maximum field RMSE was $1.303\times 10^{-10}$, and all candidate-pool optimal triplets agreed. With state, frame, and epoch differences below the defined tolerances, discrepancies in the following full-force comparison are attributed to the added force-model terms.

#### 4.5.2. High-Fidelity Validity Horizon Without Orbit Maintenance

The high-fidelity force model excluded orbit-maintenance maneuvers and was evaluated at three epochs and three target altitudes every 6 h through day 7. Days 14 and 30 were sparse diagnostics and did not enter the consecutive-failure rule. Results obtained with the uncorrected $J_{2}$ coordinates were compared with those after a causal rolling-window longitude alignment. A same-time best-fit longitude alignment, obtained from the common axial rotation that minimized the same-identity satellite-position residual, was used only to diagnose rigid phase error.

Median RMSE increased from $1.880\times 10^{-3}$ initially to $5.907\times 10^{-3}$ at day 1.5 and $7.971\times 10^{-2}$ at day 7. Table 9 summarizes the sampled validity horizons. The median horizon was 1.50 days, and the minimum was 1.25 days; the causal correction did not change any threshold-crossing time.

**Figure 16:**
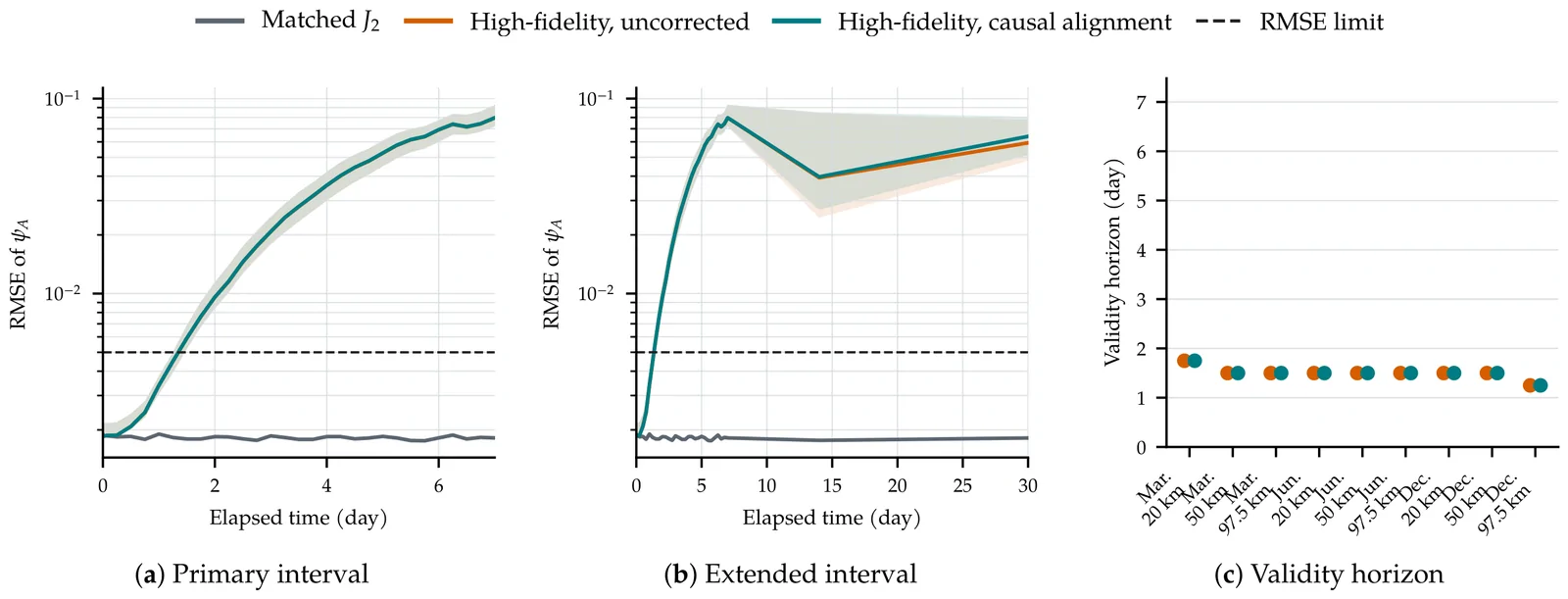
Validity under matched-$J_{2}$ and uncontrolled high-fidelity dynamics.

Figure 16 contrasts the stable matched-$J_{2}$ result with the increasing high-fidelity-model error. Bands span the 10–90th percentiles over epochs and altitudes; days 14 and 30 provide sparse long-horizon diagnostics. Alternative RMSE limits of 0.003 and 0.010 yielded median horizons of 1.00 and 1.75 days; the causal alignment did not extend these horizons.

At day 7, the same-time best-fit longitude alignment still left an RMS same-identity satellite-position residual of 450 km and a field RMSE of 0.07982, showing deformation that could not be removed by a common axial phase shift. At the March-equinox epoch, median RMSE was approximately $5.02\times 10^{-3}$ under degree/order-70 Earth gravity, with little change after successively adding solar–lunar gravity and solar radiation pressure. Adding NRLMSISE-00 drag increased the median to $2.496\times 10^{-2}$. These medians cover the primary 0–7 d samples and three target altitudes. Drag thus contributed the largest increment in this comparison for the adopted 500 km orbit and 100 kg, 1 m^2^, $C_{D}=2.2$ spacecraft model. The comparison concerns additions to the force model; central gravity was included throughout.

Orbital-element differences between the high-fidelity and matched-$J_{2}$ trajectories were compared at the onset of each sustained accuracy loss, defined by three consecutive samples. Across the nine epoch–altitude combinations, the differential RMS ranges were 0.071–0.078 km for *a*, 0.00057–$0.00305^{\circ }$ for *i*, 0.00150–$0.00217^{\circ }$ for Ω, and 0.0670–$0.0939^{\circ }$ for $u_{m}$. These deviations remained below the corresponding single-element tolerance limits in Table 7, despite the loss of field accuracy. The high-fidelity trajectories contained simultaneous differential and common-mode changes in several elements, whereas the controlled sweeps isolated individual differential perturbations. Single-element tolerance limits therefore do not ensure validity under coupled orbital drift.

#### 4.5.3. Historical Iridium Operational-Trajectory Case Study

The Iridium case study reconstructed historical operational trajectories for the 66 nominal Iridium NEXT satellites in six planes of 11 slots; one supplemental object was excluded from the primary field. Historical OMM records were obtained from the Space-Track GP_History service [33] in the CCSDS orbit data message format [34]. At each evaluation time, the most recent Orbit Mean-Elements Message (OMM) issued no later than that time was propagated from its own epoch with Simplified General Perturbations 4 (SGP4) [35], without interpolation between records or access to future records. The evaluation epoch was set to 27 May 2026. Membership, slots, and phase rates were fixed before independent training with seeds 2026, 3407, and 9011. Evaluation covered 121 samples at 6 h intervals over 30 days at 20, 50, and 97.5 km, without retraining or slot reassignment. The preanalysis protocol allowed a maximum OMM age of 78 h, equal to the 72 h sensitivity interval plus one 6 h evaluation step. All 121 samples met this limit, and 120 also met the stricter 72 h limit.

Errors were separated into neural approximation error (neural prediction versus the nominal reference field), operational-geometry error (nominal reference field versus the OMM-updated reference field), and total error (neural prediction versus the OMM-updated reference field). For every altitude and training seed, all three errors remained within the predefined accuracy thresholds throughout the 30-day window. Table 10 reports the total-error extrema.

The Iridium evaluation demonstrates that the proposed field retains the prescribed accuracy for at least 30 days on historical operational trajectories reconstructed from OMM records, establishing its long-horizon applicability to an operational Walker constellation.

### 4.6. Application Demonstration and Computational Efficiency

The application used three fixed 30 min great-circle trajectories at 50 km and 3 km/s. A library of 75 candidates combined five initial longitudes, five initial latitudes, and three headings. Using only the reference-$J_{2}$ geometry, the high-information and low-information cases minimized and maximized mean $\psi_{A}$, respectively; the repeated-boundary case maximized the triplet-transition count, with potential range used as a tie-breaker. The trajectories began 0.5 days after the March-equinox epoch, within the 1.25-day minimum sampled validity horizon. The library, selection rules, deployment time, and trigger settings were fixed before the high-fidelity results were evaluated.

For the neural policy, the estimated regret of the assigned triplet $C_{t}$ is

(43) $${\hat{\Delta \psi }}_{A}\left(t\right)=\max\left[0,\psi_{A}^{J_{2}}\left(C_{t}\right)-{\hat{\psi }}_{A}^{★}\left(t\right)\right].$$

In this policy, the neural field supplies the scalar estimate ${\hat{\psi }}_{A}^{★}\left(t\right)$; it does not predict satellite identities. The hysteretic logic compares this estimate with the reference-$J_{2}$ potential of the current assignment to determine when a search is required. A search was triggered after three consecutive samples above $\eta_{\mathrm{on}}=0.010$, subject to a 120 s minimum dwell time, or immediately after visibility loss. Rearming required the estimated regret to fall to $\eta_{\mathrm{off}}=0.004$ or below. After a trigger, the current reference-$J_{2}$ geometry was processed by the same visibility screening, range-ranked 12-satellite candidate-pool construction, and exhaustive feasible-subset search used by the reference selector. The module therefore serves as a geometric assessment and search-triggering layer, not an end-to-end satellite-selection network. Five policies—fixed, visibility-only, neural-triggered, stepwise $J_{2}$ recomputation, and high-fidelity oracle—were evaluated against a high-fidelity reference at 181 samples. Infeasible triplets were assigned a regret of 1.0, yielding the reported penalized mean regret; infeasible samples were counted separately.

The high-fidelity oracle is a non-deployable lower-bound benchmark. At each of the 181 decision times, it uses the current high-fidelity satellite positions, applies the same visibility rules and range-ranked 12-satellite candidate-pool construction, and exhaustively evaluates all feasible triplets. The minimizing triplet also defines the high-fidelity reference optimum in the regret calculation, so its regret is zero in exact arithmetic. The reported mean of $1.39\times 10^{-17}$ and maximum of $2.22\times 10^{-16}$ are floating-point round-off residuals.

**Figure 17:**
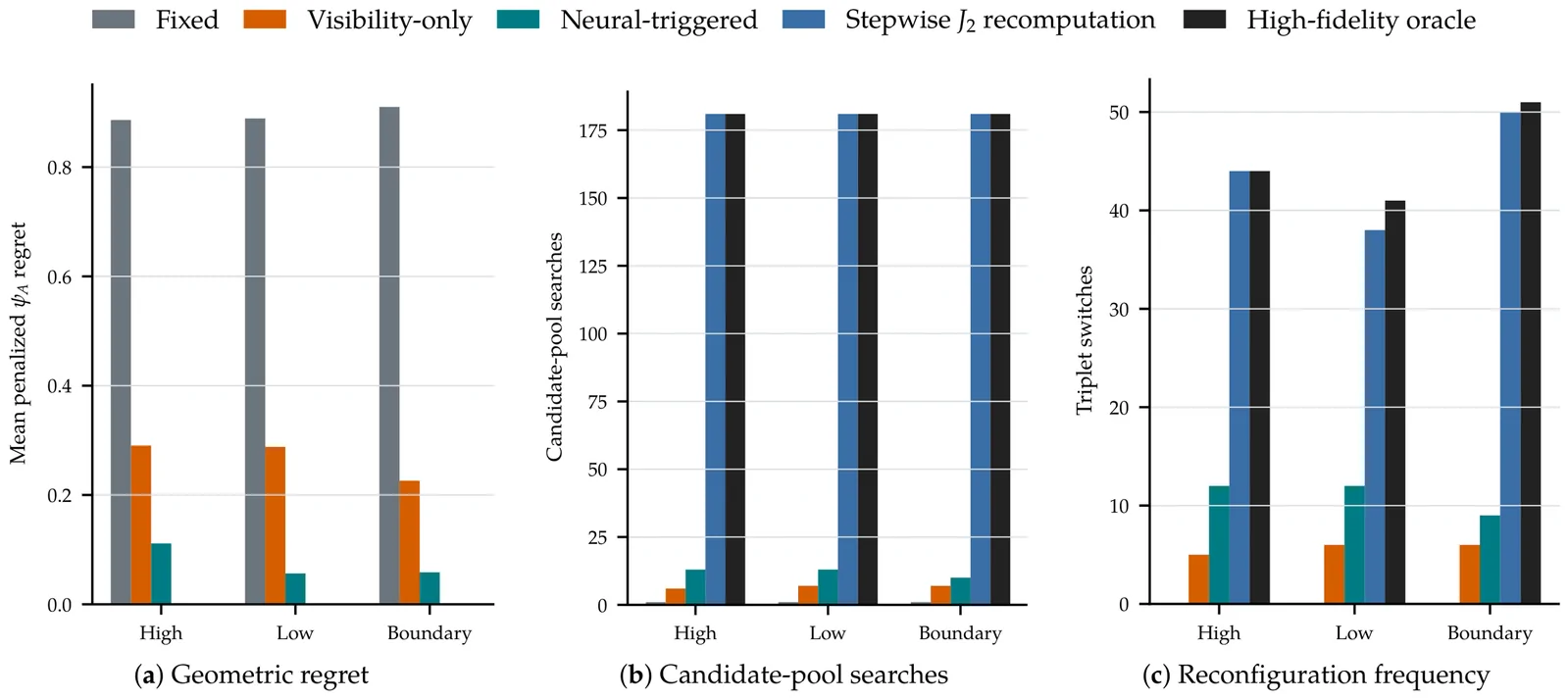
Geometric regret and reconfiguration cost of the evaluated policies.

Table 11 and Figure 17 compare the five assignment policies. Neural triggering reduced penalized mean regret by 91.6% relative to fixed assignment and 71.9% relative to visibility-only replacement, while reducing candidate-pool searches from 181 to 12. Its assigned triplet remained feasible under the high-fidelity dynamics at every sample.

Trigger agreement with the high-fidelity reference ranged from 0.492 to 0.575, and each executed change in the repeated-boundary case lowered high-fidelity regret. The observed reduction therefore represents fewer exact reference-$J_{2}$ candidate-pool searches, not direct satellite-index prediction by the neural field.

Across 27 trigger settings, penalized mean regret ranged from 0.0473 to 0.2357 with 8–18 searches, and every setting had lower penalized mean regret than the fixed and visibility-only policies. The analysis characterizes geometric reconfiguration; estimator RMSE was outside the experiment scope.

#### Computational Efficiency

Table 12 reports latency on an Intel Core i7-14700KF and NVIDIA RTX 5070 Ti; raw-coordinate timings include analytical encoding.

Relative to candidate-pool CPU evaluation, raw-coordinate inference accelerated 1000 queries by 23.4 times on CPU and 440 times on GPU; the corresponding full-grid speedups were 17.5 and 5567. A single GPU query was slower than candidate-pool CPU evaluation because launch overhead dominated. Likewise, reducing candidate-pool searches by 92.8–94.5% yielded only a 1.2–2.9% sequential CPU latency reduction when features were rebuilt at every sample. Batched field generation and repeated many-point evaluation provided the principal computational benefit; isolated queries provided little acceleration.

## 5. Discussion

The field applies when a finite constellation permutation is identified, the target altitude lies within the trained interval, and the operational relative geometry remains close to that represented in the training data. The controlled one-element scans provide configuration-specific screening intervals rather than joint drift guarantees or orbit-maintenance thresholds. Under uncontrolled full-force propagation without maintenance, the median and minimum sampled validity horizons were 1.50 and 1.25 d, whereas the Iridium evaluation retained the prescribed field accuracy for at least 30 d on reconstructed historical operational trajectories. Satellite loss, slot reassignment, constellation reconfiguration, or geometry-altering maneuvers require an updated reference constellation and renewed verification of the symmetry relation and surrogate accuracy. The neural field provides differentiable geometric queries and search triggering, while exact candidate-pool search determines satellite identities when reassignment is required. The current sensing model omits field-of-view constraints, outages, timing errors, and recursive estimation, and $\psi_{A}$ does not directly predict tracking RMSE. Future work will address efficient field adaptation under configuration changes, ephemeris corrections, low-dimensional constellation-deformation variables, and estimator-level tasking and trajectory planning.

## 6. Conclusions

This study introduced a symmetry-reduced differentiable angle-only information field. Standard Walker parameterization and central-gravity-plus-$J_{2}$ dynamics define the nominal constellation model, while the proposed representation maps axial and finite-permutation symmetries to bounded co-precessing and fast-phase coordinates. The bounded representation reduced RMSE relative to a matched two-body encoding; independently, neural altitude gradients agreed closely with centered finite differences. Finite-symmetry closure was verified for Delta, Star, and Rosette constellations. The field remained accurate for 30 d under matched $J_{2}$, reached a minimum sampled validity horizon of 1.25 d under uncontrolled full-force propagation, and retained the prescribed accuracy for at least 30 d on historical Iridium operational trajectories. Field-guided triggering reduced candidate-pool searches by 93.4%, while exact search continued to determine satellite identities. The resulting framework provides a differentiable and computationally efficient geometric layer for persistent sensor selection with monitored field validity.

## Figures and Tables

**Table 1:** Neural information-field architecture.

Stage	Input Dimension	Hidden Mapping	Output Dimension
Reduced phase-context encoder	20	4×256 SiLU	128; LayerNorm
Information backbone	132	4×256 SiLU	256; SiLU
Output branches	256	Two linear maps	1+1

Note: L×W denotes *L* hidden layers of width *W*.

**Table 2:** Common experimental configuration.

Component	Parameter	Value
Constellation	Walker Delta arrangement	25 planes × 25 satellites, F=1
Constellation	Inclination; satellite orbital altitude	$86^{\circ }$; 500 km
Earth model	$R_{E}$; μ; $J_{2}$	6378.137 km; 398,600.4418 km^3^/s^2^; 0.00108262668
Earth model	Earth rotation rate $\omega_{E}$	$7.2921159\times 10^{-5}$ rad/s
Relative-periodic orbit	$T_{\mathrm{rp}}$; $\Delta t_{\mathrm{slot}}$; ${\dot{\Omega }}_{\mathrm{rp}}$	5676.9223 s; 227.0769 s; $-1.08136\times 10^{-7}$ rad/s
Orbit integration	Solver; (rtol,atol); maximum step	DOP853; $\left(10^{-11},10^{-13}\right)$; 10 s
Angle sensor	Angular standard deviation	10 arcsec per angular component
Availability	Maximum range; minimum Earth clearance	2800 km; 5 km
Subset search	Candidate pool; $N_{\mathrm{sel}}$	Top 12 individual candidates; 3 satellites
FIM numerics	Reciprocal-condition threshold	$10^{-11}$
Field domain	Longitude–latitude grid; altitude interval	$1^{\circ }\times 1^{\circ }$; 15–100 km
Neural field	Hidden layers per stage; width; latent dimension	4; 256; 128
Neural field	Periodic harmonics; trainable parameters	1–4; 533,634
Optimization	Optimizer; learning rate; batch size; epochs	AdamW; $10^{-3}$; 32,768; 1500
Optimization	Loss	Huber regression +0.1 binary-feasibility loss
External propagation	Engine; license	TudatPy 1.0.0; BSD-3-Clause
External propagation	Frame; time coordinate	Earth/J2000; seconds since J2000 in TDB
External propagation	Integrator; maximum step	Variable-step RKF7(8); 60 s
High-fidelity force model	Earth gravity; perturbations	GOCO05c degree/order 70; solar/lunar gravity, SRP, NRLMSISE-00 drag

**Table 3:** Relative-periodic $J_{2}$ closure diagnostics.

Diagnostic	Adjacent-Slot Relation	Full-Period Closure
Position RMS (km)	$3.668\times 10^{-12}$	$9.704\times 10^{-10}$
Velocity RMS (km/s)	$8.137\times 10^{-14}$	$9.249\times 10^{-13}$
Relative energy span	$4.289\times 10^{-15}$	
Normalized boundary-condition residual	$5.283\times 10^{-14}$	

**Table 4:** Representation ablation on unseen altitude–phase samples.

Metric	TB	J2-SR	J2-SR Worst
Global RMSE	$7.664\times 10^{-3}\pm 1.046\times 10^{-2}$	$1.154\times 10^{-3}\pm 3.141\times 10^{-4}$	$1.445\times 10^{-3}$
Poor-geometry RMSE	$8.051\times 10^{-3}\pm 1.084\times 10^{-2}$	$1.404\times 10^{-3}\pm 3.496\times 10^{-4}$	$1.728\times 10^{-3}$
95th percentile $|e_{\psi }|$	$1.666\times 10^{-2}\pm 2.403\times 10^{-2}$	$2.341\times 10^{-3}\pm 6.383\times 10^{-4}$	$2.929\times 10^{-3}$
99th percentile $|e_{\psi }|$	$2.435\times 10^{-2}\pm 3.354\times 10^{-2}$	$3.534\times 10^{-3}\pm 8.688\times 10^{-4}$	$4.303\times 10^{-3}$
Maximum altitude-group RMSE	$8.953\times 10^{-3}\pm 1.227\times 10^{-2}$	$1.321\times 10^{-3}\pm 3.586\times 10^{-4}$	$1.653\times 10^{-3}$
Maximum phase-group RMSE	$1.029\times 10^{-2}\pm 1.227\times 10^{-2}$	$1.259\times 10^{-3}\pm 3.143\times 10^{-4}$	$1.525\times 10^{-3}$
$R^{2}$	0.97794±0.03739	0.999766±0.000119	0.999650

Note: Entries are the mean ± sample standard deviation over three training seeds. “Worst” denotes the largest error, or the smallest $R^{2}$, among the three seeds.

**Table 5:** Approximation near visibility-set and subset-selection transitions.

Event	Region	RMSE	MAE	P95 |*e*|
Visibility set	Boundary	1.335 [0.868, 1.699]	0.928 [0.608, 1.107]	2.692 [1.574, 3.314]
Visibility set	Off-event neighborhood	1.495 [0.784, 1.977]	1.054 [0.592, 1.360]	2.978 [1.653, 3.733]
Optimal subset	Boundary	2.479 [1.617, 3.232]	2.075 [1.390, 2.754]	4.791 [2.964, 5.940]
Optimal subset	Off-event neighborhood	1.509 [1.046, 1.981]	1.157 [0.786, 1.554]	2.976 [1.894, 4.079]

Note: All values are in units of $10^{-3}$ and give the mean [minimum, maximum] over three training seeds. Each seed-wise metric pools samples from 30 events; the bracketed ranges are not confidence intervals.

**Table 6:** Constellation-parameter sensitivity of the reference and neural fields.

Configuration	Median Reference $\psi_{A}$	Median Visible Satellites	Neural RMSE	Worst-Seed RMSE	Worst-Seed $R^{2}$
Baseline: 25×25, 500 km, $86^{\circ }$, F=1	−1.3101	26	0.001154 (0.000314)	0.001445	0.999650
20×20	−1.2548	17	0.001137 (0.000223)	0.001395	0.999844
30×30	−1.3617	37	0.001603 (0.000299)	0.001851	0.999153
$i=70^{\circ }$	−1.2921	32	0.001456 (0.000172)	0.001654	0.999881
$i=78^{\circ }$	−1.3015	27	0.001356 (0.000056)	0.001410	0.999773
$h_{s}=400$ km	−1.3472	26	0.001709 (0.000463)	0.002108	0.999585
$h_{s}=600$ km	−1.2701	25	0.001202 (0.000199)	0.001321	0.999468
F=0	−1.3066	26	0.002049 (0.000482)	0.002581	0.998838
F=12	−1.3177	26	0.003476 (0.002473)	0.006275	0.993005

Note: Unlisted parameters retain their baseline values. Neural RMSE is the three-seed mean (sample standard deviation).

**Table 7:** Sampled transition intervals under single-element orbital drift.

Orbital Element	Largest Sampled Amplitude Within Tolerance	First Sampled Amplitude Exceeding Tolerance
Semi-major axis, *a*	9 km	10 km
Inclination, *i*	$0.225^{\circ }$	$0.250^{\circ }$
RAAN, Ω	$0.200^{\circ }$	$0.225^{\circ }$
Mean argument of latitude, $u_{m}$	$0.125^{\circ }$	$0.150^{\circ }$

Note: One orbital element was perturbed in each test. Values are satellite-wise RMS amplitudes determined by the worst result over all perturbation patterns, training seeds, phases, and target altitudes; no interpolation was applied.

**Table 8:** Matched-$J_{2}$ propagation and field-consistency checks.

Layer	Diagnostic	Observed	Predefined Threshold
Reference $J_{2}$, $2^{\circ }$	Maximum time-resolved field RMSE	$1.581\times 10^{-3}$	<0.005
Reference $J_{2}$, $2^{\circ }$	Minimum time-resolved $R^{2}$	0.999578	>0.99
Reference $J_{2}$, targeted $1^{\circ }$	Maximum field RMSE	$1.695\times 10^{-3}$	<0.005
Matched-$J_{2}$ consistency	Maximum position difference (km)	$4.632\times 10^{-7}$	<$5\times 10^{-3}$
Matched-$J_{2}$ consistency	Maximum velocity difference (km/s)	$5.132\times 10^{-10}$	<$5\times 10^{-6}$
Matched-$J_{2}$ consistency	Maximum subpoint-longitude RMS (deg)	$1.026\times 10^{-8}$	<$10^{-5}$
Matched-$J_{2}$ consistency	Maximum field RMSE	$1.303\times 10^{-10}$	<$10^{-5}$
Matched-$J_{2}$ consistency	Minimum field $R^{2}$	1.000000	>0.999999

**Table 9:** High-fidelity validity horizons without orbit maintenance.

Absolute Epoch	Target Altitude (km)	Raw	Causal Shift
March equinox	20.0	1.75	1.75
March equinox	50.0	1.50	1.50
March equinox	97.5	1.50	1.50
June solstice	20.0	1.50	1.50
June solstice	50.0	1.50	1.50
June solstice	97.5	1.50	1.50
December solstice	20.0	1.50	1.50
December solstice	50.0	1.50	1.50
December solstice	97.5	1.25	1.25

Note: Horizons are in days and follow Equation (42) on the regular 6 h grid.

**Table 10:** Historical Iridium operational-trajectory accuracy and validity results.

Training Seed	Maximum Total RMSE	Minimum Total $R^{2}$	Sampled Validity Horizon
2026	0.004089	0.999048	≥30 days
3407	0.004359	0.998916	≥30 days
9011	0.004199	0.998994	≥30 days

**Table 11:** Reconfiguration policy comparison.

Policy	Penalized Mean Regret	Candidate-Pool Searches	Switches	Infeasible Samples
Fixed	0.8951	1.00	0.00	460
Visibility-only	0.2683	6.67	5.67	1
Neural-triggered	0.07545	12.00	11.00	0
Stepwise $J_{2}$ recomputation	$9.86\times 10^{-6}$	181.00	44.00	0
High-fidelity oracle	$1.39\times 10^{-17}$	181.00	45.33	0

Note: Regret, searches, and switches are means over the three trajectories; infeasible samples are totals over all 543 trajectory–time evaluations. Infeasible triplets were assigned a regret of 1.0.

**Table 12:** Query latency and speedup.

Method	Hardware	1 Query	1000 Queries	65,341 Queries	Speedup
Candidate-pool reference	CPU	0.779 (0.009)	512.879 (1.109)	31,877.518 (342.331)	1.00
Neural, raw coordinates	CPU	0.481 (0.007)	21.949 (0.022)	1823.551 (15.920)	17.48
Neural, pre-encoded	CPU	0.253 (0.003)	21.532 (0.014)	1809.791 (5.393)	17.61
Neural, raw coordinates	GPU	1.111 (0.004)	1.166 (0.006)	5.727 (0.774)	5566.64
Neural, pre-encoded	GPU	0.528 (0.005)	0.573 (0.006)	5.213 (0.858)	6115.50

Note: Latencies are median (IQR) values in milliseconds over 30 repetitions. Full-grid speedup is relative to candidate-pool CPU evaluation of the same 65,341 points.

## Data Availability

The derived data, model checkpoint, and code supporting the findings of this study are available on Zenodo at https://doi.org/10.5281/zenodo.21730757 (accessed on 15 September 2026) and in the public GitHub repository at https://github.com/Hengguo-Zhang/neural-information-field (accessed on 15 September 2026). Raw Space-Track records are not redistributed because their use is subject to the data provider’s terms. The repository provides instructions for authorized retrieval and includes the derived data used in the reported analyses.

## Abbreviations

The following abbreviations are used in this manuscript:

CPU	Central processing unit
FIM	Fisher information matrix
GPU	Graphics processing unit
IQR	Interquartile range
J2-SR	$J_{2}$ symmetry-reduced encoding
LEO	Low Earth orbit
MAE	Mean absolute error
OMM	Orbit mean-elements message
RAAN	Right ascension of the ascending node
RMS	Root mean square
RMSE	Root-mean-square error
SGP4	Simplified general perturbations 4
SiLU	Sigmoid linear unit
SRP	Solar radiation pressure
TB	Matched two-body encoding
TDB	Barycentric Dynamical Time

## References

[1] D’Anniballe A., Felicetti L., Hobbs S.E. (2025). Preliminary Analysis and Design of an Optical Space Surveillance and Tracking Constellation for LEO Coverage. Acta Astronaut..

[2] Perez A.C., Geller D.K., Lovell T.A. (2018). Non-Iterative Angles-Only Initial Relative Orbit Determination with *J*_2_ Perturbations. Acta Astronaut..

[3] An Q., Yuan Y., Zhang Z., Liu H., Yue X. (2024). On the Information Evolution and Sensor Deployment for Multi-Spacecraft Cooperative Visual 3-D Sensing of Orbital Targets. IEEE Sens. J..

[4] Greaves J., Scheeres D. (2024). Spacecraft to Spacecraft Absolute Tracking for Autonomous Navigation of a Distributed Space System from Relative Sensors. J. Astronaut. Sci..

[5] Dogancay K., Hmam H. (2024). UAV Path Optimization for Angle-Only Self-Localization and Target Tracking Based on the Bayesian Fisher Information Matrix. Sensors.

[6] Cefai E.-J., Coombes M., O’Boy D. (2025). Intelligent Wireless Sensor Network Sensor Selection and Clustering for Tracking Unmanned Aerial Vehicles. Sensors.

[7] Joshi S., Boyd S. (2009). Sensor Selection via Convex Optimization. IEEE Trans. Signal Process..

[8] Chepuri S.P., Leus G. (2015). Sparsity-Promoting Sensor Selection for Non-Linear Measurement Models. IEEE Trans. Signal Process..

[9] Xue C., Cai H., Gehly S., Jah M., Zhang J. (2024). Review of Sensor Tasking Methods in Space Situational Awareness. Prog. Aerosp. Sci..

[10] Siew P.M., Jang D., Roberts T.G., Linares R. (2022). Space-Based Sensor Tasking Using Deep Reinforcement Learning. J. Astronaut. Sci..

[11] Fedeler S., Holzinger M., Whitacre W. (2022). Sensor Tasking in the Cislunar Regime Using Monte Carlo Tree Search. Adv. Space Res..

[12] Peng H., Li J., Tian J., Wang Y. (2023). A Game Theoretic Self-Organization for Satellite-Based Optical Sensor Allocation. Aerosp. Sci. Technol..

[13] Shimane Y., Ho K. (2025). Multi-Sensor Tasking for Ground-Based Space Situational Awareness via Job-Shop Scheduling Problem. J. Astronaut. Sci..

[14] Patel M., Tomita K., Ho K. (2025). Concurrent Optimization of Satellite Phasing and Tasking for Cislunar Space Situational Awareness. J. Astronaut. Sci..

[15] Mencarelli L., Floquet J., Georges F. (2024). Matheuristics Approaches for the Satellite Constellation Design Problem. Optim. Eng..

[16] Zhang Z., Scaramuzza D. Beyond Point Clouds: Fisher Information Field for Active Visual Localization. Proceedings of the 2019 International Conference on Robotics and Automation (ICRA).

[17] Chang J., Duquette R., Thai K., Zhou T., Pham M., Lin V., Wood K. Combining Genetic Algorithms and Machine Learning for Exploring the Navigation Satellite Constellation Design Tradespace. Proceedings of the 33rd International Technical Meeting of the Satellite Division of the Institute of Navigation (ION GNSS+ 2020).

[18] Xie Y., Takikawa T., Saito S., Litany O., Yan S., Khan N., Tombari F., Tompkin J., Sitzmann V., Sridhar S. (2022). Neural Fields in Visual Computing and Beyond. Comput. Graph. Forum.

[19] Tancik M., Srinivasan P.P., Mildenhall B., Fridovich-Keil S., Raghavan N., Singhal U., Ramamoorthi R., Barron J.T., Ng R. (2020). Fourier Features Let Networks Learn High Frequency Functions in Low Dimensional Domains. Adv. Neural Inf. Process. Syst..

[20] Sitzmann V., Martel J.N.P., Bergman A.W., Lindell D.B., Wetzstein G. (2020). Implicit Neural Representations with Periodic Activation Functions. Adv. Neural Inf. Process. Syst..

[21] Duvall J., Duraisamy K. (2025). Discretization-Independent Surrogate Modeling of Physical Fields around Variable Geometries Using Coordinate-Based Networks. Data-Centric Eng..

[22] Raissi M., Perdikaris P., Karniadakis G.E. (2019). Physics-Informed Neural Networks: A Deep Learning Framework for Solving Forward and Inverse Problems Involving Nonlinear Partial Differential Equations. J. Comput. Phys..

[23] Karniadakis G.E., Kevrekidis I.G., Lu L., Perdikaris P., Wang S., Yang L. (2021). Physics-Informed Machine Learning. Nat. Rev. Phys..

[24] Hou Z., Sun Q., Dang Z. (2025). Neural Networks-Based Solution of the Two-Body Problem. Astrodynamics.

[25] Mahmud M.S., Jiang Y., Liu R., Zhou Z., Xu B. (2026). Physics-Informed Neural Network for Predicting Orbital Parameters of Low Earth Orbit Satellites Using Two Line Element Dataset. Adv. Space Res..

[26] Walker J.G. (1984). Satellite Constellations. J. Br. Interplanet. Soc..

[27] Ma Y., He Y., Xu M., Zheng Y. (2022). Global Searches of Frozen Orbits around an Oblate Earth-Like Planet. Astrodynamics.

[28] Vallado D.A. (2013). Fundamentals of Astrodynamics and Applications.

[29] Ballard A.H. (1980). Rosette Constellations of Earth Satellites. IEEE Trans. Aerosp. Electron. Syst..

[30] Kumar K., van Barneveld P.W.L., Dirkx D., Melman J.C.P., Mooij E., Noomen R. TUDAT: A Modular and Robust Astrodynamics Toolbox. Proceedings of the 5th International Conference on Astrodynamics Tools and Techniques (ICATT).

[31] Fecher T., Pail R., Gruber T. (2017). GOCO05c: A New Combined Gravity Field Model Based on Full Normal Equations and Regionally Varying Weighting. Surv. Geophys..

[32] Picone J.M., Hedin A.E., Drob D.P., Aikin A.C. (2002). NRLMSISE-00 Empirical Model of the Atmosphere: Statistical Comparisons and Scientific Issues. J. Geophys. Res. Space Phys..

[33] United States Space Command Space-Track.org Documentation: General Perturbations and GP History. https://www.space-track.org/documentation.

[34] Consultative Committee for Space Data Systems (2023). Orbit Data Messages.

[35] Vallado D.A., Crawford P., Hujsak R., Kelso T.S. Revisiting Spacetrack Report No. 3. Proceedings of the AIAA/AAS Astrodynamics Specialist Conference and Exhibit.

